# Triple-FRET multi-purpose fluorescent probe for three-protease detection†

**DOI:** 10.1039/d2ra05125g

**Published:** 2022-10-10

**Authors:** David Milićević, Jan Hlaváč

**Affiliations:** Department of Organic Chemistry, Faculty of Science, Palacký University Olomouc 17. Listopadu 12 771 46 Olomouc Czech Republic jan.hlavac@upol.cz

## Abstract

A new, robust and reliable methodology for three-protease screening in a single-enzyme mode has been developed and verified, employing a multi-purpose peptide probe with three selectively cleavable sites furnished with four fluorophores. A triple-FRET-based single-excitation quadruple-emission concept for unambiguous sensing of trypsin, chymotrypsin and caspase-8 in the lowest detectable concentrations of 0.5 ng mL^−1^, 0.2 μg mL^−1^, and 2 U mL^−1^, respectively, has been applied and graphically depicted. Then the developed 4-dye probe has been also studied from the perspective of simultaneous two-protease screening, which was found only partially feasible, primarily due to unselective chymotrypsin cleavage.

## Abstract 2

A triple-FRET four-dye system for detection of three proteases has been developed and verified.

## Graphical abstract

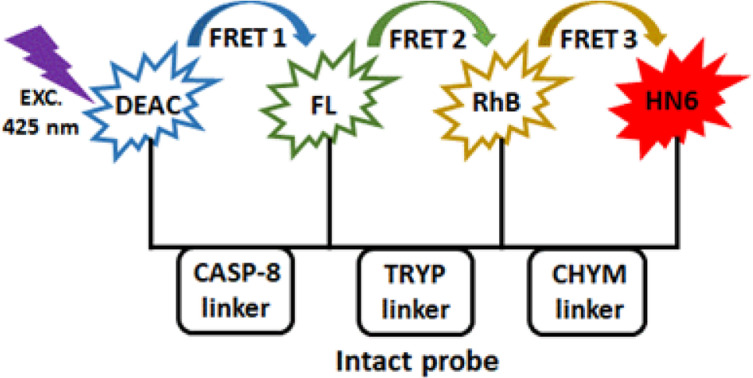

## 1. Introduction

With the growing interest in sensing proteolytic activities and studying their tight correlations with some pathological processes, numerous papers included in a few comprehensive reviews were published in the first decade of the current century. Various proteases were found indispensable in different fields such as degradomics,^1^ signalling pathways,^2^ and drug targeting.^3^ Around the same time, another review consolidating Förster resonance energy transfer (FRET) as a powerful tool from the perspective of enzyme kinetics and activity studies has been issued.^4^ Based on the effectiveness of fluorescence energy transfer between neighbouring fluorescent dyes, significant changes in molecular structures (caused by, *e.g.* enzymatic cleavage) or solely in their conformations could be followed in real-time.^5^

Until nowadays, several applications employing FRET principles for the purpose of miscellaneous analytes' screening have been reported in various scientific areas, including chemistry,^6^ biology,^7^ pharmacology,^8^ and biomedicine.^9^ Although many single-FRET probes are described in the literature, multi-FRET systems for detecting different enzyme species are pretty rare and appear only sporadically. Two examples of fluorophore-furnished linear peptide-based multi-FRET sensors capable of sequential^10^ or simultaneous^11^ detection of caspase-3 and MMP-2 have been reported by Zhang's research group. Similarly, a dual-FRET ratiometric system for synchronous screening of trypsin and chymotrypsin has been developed in our group.^12^ In addition, Bradley with co-workers have constructed a double-FRET 4-dye dual substrate for selective and simultaneous detection of two human lung cancer-relevant proteases, namely thrombin and MMP.^13^ Finally, synchronous sensing of two proteases (M^pro^ and PL^pro^), tightly related to the SARS-CoV-2 replication cycle, has been performed *in vitro* as well as in cells.^14^ For this purpose, a peptide-based substrate equipped with two fluorescent dyes and a black hole quencher has been utilized.

As an efficient alternative to conventional multi-fluorophore linear energy transfer, various FRET network approaches,^15^ including concentric FRET (cFRET), have been gaining popularity in recent years. A core of such a cFRET system is most commonly represented by semiconductor quantum dots that also frequently perform the functions of peptide attaching sites and FRET donors.^16^ Their potential has already been proven in a few studies dealing with a single protease screening,^17,18^ as well as parallel sensing of two or more enzyme species.^19,20^ On the other hand, some drawbacks and disadvantages of the cFRET QD-based methodology, including time-consuming calibration and evaluation processes, have been reported as well.^21,22^

Even though some innovative enzyme screening techniques based on FRET networks^15^ have been found superior from imaging, bioanalysis, and quantitative biological methods points of view, linear peptide FRET systems are still indispensable in numerous applications and are extensively used in various assay kits for protease detection. Their relatively facile synthesis and straightforward applicability do not require sophisticated equipment or expensive materials, making conventional peptide-based protease detection accessible to laboratories around the world.

Herein, we report a multi-purpose linear triple-FRET peptide probe for reliable detection of three model proteases. In the presence of target enzyme species, the corresponding recognizable sites of the substrate are cleaved, resulting in the disruption of a FRET sequence. Based on consequent notable changes in fluorescence responses of particular fluorophores, the existence of individual proteases in a system could be determined. The developed methodology has been evaluated, verified, and proven highly tolerable towards impurities in a sample.

## 2. Methods and materials

**Fig. 1:**
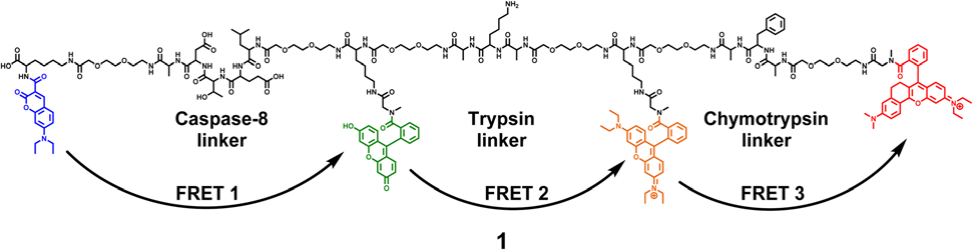
Four-dye probe (1) decorated with coumarin (blue), fluorescein (green), Rhodamine B (yellow) and HN6 dye (red) – the substrate for caspase-8, trypsin and chymotrypsin screening.

The 4-dye probe (1) (Fig. 1) was synthesized on polystyrene Wang resin (0.9 mmol g^−1^, AAPPTec, Louisville, KY, USA), applying moderately adapted multi-step solid-phase peptide synthesis. The chemicals and solvents were obtained from the available commercial sources, while some fluorophores, namely 7-(diethylamino)coumarin-3-carboxylic acid (DEAC)^23^ and HN6,^24^ were synthesized according to literature procedures.

### 2.1. Synthesis, purification, and storage

Polystyrene Wang resin (500 mg) was placed into a plastic syringe (B. Braun Melsungen AG, Melsungen, Germany) equipped with a sintered plastic filter (Torviq, Tucson, AZ, USA), subsequently prewashed with dichloromethane (5 times), and reacted with appropriate reagents in suitable concentrations. The reaction mixture was shaken on a microplate shaker (Thermo Fisher Scientific, Waltham, MA, USA). Then the solid support was manually washed with dimethylformamide (10 times) and dichloromethane (10 times). The synthetic scheme with the specified reaction conditions is presented in (ESI – Scheme S1†).

To analyze an intermediate after each reaction step, a small amount of a resin-bound peptide was chemically cleaved from a solid support, using 50% TFA in DCM. After evaporation of volatiles, the residuum was diluted with acetonitrile/water 1 : 1 (v/v) and analyzed on UHPLC chromatograph (Acquity) with photodiode array detector and a single quadrupole mass spectrometer (Waters, Borehamwood, UK), employing C-18 XSelect HSS T3 2.5 μm XP (50 × 3.0 mm) column (Waters, Borehamwood, UK). As a mobile phase, ammonium acetate (10 mM) in ultrapure water and acetonitrile (gradient 20–80% during the first 4.5 min or 50–80% during the first 3 min) were used. The chromatograms and corresponding mass fragmentation profiles can be found in (ESI – Fig. S1–S5†).

The resin-immobilized final crude product was treated with a cleavage cocktail (50% TFA in DCM) for 60 min. The volatiles were removed under a stream of nitrogen. Sticky colourful material was diluted with acetonitrile/0.1% TFA in ultrapure H_2_O 2 : 1 (v/v) and purified on a semi-prep HPLC column (Aeris 5 μm 150 × 21.2 mm peptide XB-C18 100 Å, Phenomenex, California, USA), using a gradient of 20–80% acetonitrile in 0.1% TFA in ultrapure H_2_O within 20 min. The flow rate was set to 15 mL min^−1^. The combined fractions were concentrated *in vacuo* (Buchi R-215 Rotavapor, Marshall Scientific, Flawil, Switzerland), and freeze-dried for 48 hours (Scanvac Coolsafe Freeze Dryer, LaboGene, Lillerød, Denmark) to obtain a dark blue powder that was afterwards stored at −80 °C in a deep freezer (Arctiko, Esbjerg Kommune, Denmark).

### 2.2. Compound characterization and stability testing

The isolated 4-dye substrate (1) was characterized by LC-MS analysis and HRMS. Its final purity was determined based on LC-MS analysis, testing different analytical columns, mobile phases, gradients and elution times (ESI – Fig. S6 and S7†). For stability testing, the 4-dye probe (1) in the form of powder (stored at −80 °C) was dissolved in an appropriate volume of Tris–HCl buffer (pH = 8.0) to obtain a 50 μM solution and subsequently analyzed by LC-MS. This process was repeated a few times over 30 days.

### 2.3. Proteases and buffer solution

Trypsin (bovine pancreas, TPCK treated, ≥10 000 BAEE units per mg protein) and α-chymotrypsin (bovine pancreas, TLCK treated, type VII, ≥40 units per mg protein) were ordered from Sigma-Aldrich (Steinheim, Germany). Caspase-8 (human, recombinant, active, 5000 units per mg protein) was provided by Enzo Life Sciences, Inc. (New York, USA). All enzyme species were obtained in the form of lyophilized white to off-white powders. They were reconstituted according to the providers' instructions, aliquoted in Eppendorf tubes, and finally stored at −80 °C in a deep freezer (Arctiko, Esbjerg Kommune, Denmark).

Enzyme assays were carried out in 0.1 M Tris–HCl buffer solution in ultrapure water, with the addition of NaCl (25 mM), EDTA (5 mM) and glycerol (2.5% v/v). The pH of the media (pH = 8.0) was adjusted by gradual dropwise addition of concentrated NaOH aqueous solution.

### 2.4. Single protease detection

The appropriate amount of the 4-dye probe (1) was dissolved in 950 μL of Tris–HCl buffer. The resulting solution was placed into a plastic fluorimeter cuvette and preheated to 37 °C. Afterwards, the fluorescence emission spectrum upon excitation with the light source of 425 nm was measured at the time 0 using a fluorescence spectrometer (Cary Eclipse, Agilent Technologies, Santa Carla, CA, USA). Then 20 μL of 1 mM HCl with/without enzyme (trypsin or chymotrypsin) and 30 μL of physiological saline solution with/without enzyme (caspase-8) were added, resulting in a final volume of 1 mL of 5 μM 4-dye substrate (1). In the case of caspase-8, dl-dithiothreitol (10 mM) was also present in a buffer. Fluorescence emission responses were then repeatedly measured at the times 2, 5, 8, 12, 16, 20, 25, 30, 35, 40, and 45 min. During the entire period of the experiment, a fluorimeter cuvette was placed in a cell holder preheated to 37 °C, and located inside a fluorescence spectrometer.

### 2.5. Simultaneous screening of two proteases

A slightly modified experimental procedure for a single enzyme detection was also utilized for simultaneous sensing of two proteases. After an appropriate quantity of 4-dye probe (1) was dissolved in 950 μL of Tris–HCl buffer solution and subsequently preheated to 37 °C, 20 μL of 1 mM HCl and 30 μL of physiological saline solution with an appropriate couple of studied proteases were added into a sample. A fluorescence emission spectra were then measured at 2, 5, 8, 12, 16, and 20 min upon excitation with 425 nm. Promptly afterwards, 50 μL of DTT (200 mM) in Tris–HCl buffer was added to the incubated solution, and fluorescence response was then repeatedly measured every 5 min (at times 25, 30, 35, 40, and 45 min of an experiment).

### 2.6. Spectral and photophysical properties

The fluorescence intensity readouts for the 4-dye probe in Tris–HCl buffer were performed at the corresponding emission maxima (480 nm, 522 nm, 595 nm, and 665 nm) upon uniform excitation at 425 nm. The observed values were found comparable with the emission maxima of isolated fluorophores, DEAC (*λ*_EMS_ = 474 nm; *λ*_EXC_ = 446 nm), FL (*λ*_EMS_ = 514 nm; *λ*_EXC_ = 492 nm), RhB (*λ*_EMS_ = 579 nm; *λ*_EXC_ = 556 nm), and HN6 (*λ*_EMS_ = 641 nm; *λ*_EXC_ = 602 nm), measured in Tris–HCl buffer. The quantum yields of the individual fluorophores attached to the peptide backbone of the target substrate were calculated using the general formula *Φ*_X_ = *Φ*_Ref_ × (∇_X_/∇_Ref_) × (*η*_X_^2^/*η*_Ref_^2^), where *Φ* denotes fluorescence quantum yield, ∇ represents a gradient of integrated fluorescence intensity *vs.* absorbance and *η* is a solvent refractive index. As the reference fluorophores, fluorescein (0.1 M NaOH), Rhodamine 6G (water) and Rhodamine B (water) were utilized. FRET efficiencies (*E*) for each of the three FRET couples, namely DEAC–FL, FL–RhB and RhB–HN6, were obtained by employing the general formula *E* = *F*_A_/(*F*_A_ + *F*_D_), where *F*_A_ represents the integrated fluorescence emission response of an acceptor upon donor excitation and *F*_D_ denotes integrated fluorescence emission response of a donor upon donor excitation. The measured integrated fluorescence emission responses for individual acceptors in FL–RhB and DEAC–FL pairs were corrected in accordance with calculated FRET efficiencies between RhB–HN6 and FL–RhB, respectively. Direct excitations of individual acceptors with corresponding donor excitation wavelengths were found negligible and were not considered in calculations. Afterwards, a total FRET efficiency for the whole triple-FRET 4-dye probe sequence was obtained by multiplying calculated FRET efficiencies for all three individual FRET pairs. The numerical data are collected in (ESI – Tables S1 and S2†).

## 3. Results and discussion

A triple FRET probe for detecting three model proteases, caspase-8, trypsin, and chymotrypsin, consists of a peptide backbone with three selectively cleavable sites decorated with four fluorophores (Fig. 1).

Each of these three protease linkers is on both sides prolonged with a polyethylene glycol-based (PEG) spacer that facilitates the approachability of individual proteases towards their target peptide sequences. Upon excitation of coumarin with the light source of 425 nm, the energy is transferred through fluorescein (FRET 1) and Rhodamine B (FRET 2) to HN6 (FRET 3), resulting in relatively low emission intensities of the first three dyes and predominant fluorescence response of HN6. In the presence of a particular enzyme species, an appropriate peptide sequence is disrupted, and a corresponding FRET transfer is interrupted as well. Consequently, a notable increase in fluorescence intensity of at least one fluorescent dye is observed. The graphical presentation of proteases' activities towards the 4-dye substrate and corresponding FRET transfers can be found in (ESI – Fig. S12†).

Based on the above described 4-dye probe (1) characteristics, our primary goal was to develop a model for three protease detection in a single-enzyme mode using one uniform excitation. At the same time, we considered the possibility of distinguishing among three pairs of proteases, and in the best-case scenario, among all seven theoretically possible combinations of three enzymes in a mixture.

### 3.1. Synthetic approach

The construction of the 4-dye probe (1) is depicted in (ESI – Scheme S1†). It is based on solid-phase organic synthesis, starting with introducing Fmoc-Lys(Dde)-OH to the Wang resin in the first reaction step to afford Resin 1. Afterwards, DEAC was bound *via* an amide bond as the first fluorophore, continuing with the subsequent removal of the Dde protecting group from lysine, and gradually building a linear peptide strain using multi-step solid-phase peptide synthesis. During this process, three different types of Fmoc-protected lysine compounds were employed. Starting with Fmoc-Lys(Dde)-OH, its sidechain was later used as an anchoring site for fluorescein. Then Fmoc-Lys(Boc)-OH was utilized as a centre of the Ala-Lys-Ala trypsin cleavage sequence. Finally, Fmoc-Lys(Mtt)-OH served as a hook for the Rhodamine B attachment. Based on our previous experience, three out of four utilized fluorophores, namely fluorescein, Rhodamine B and HN6, were found troublesome from the perspective of their binding to a solid support, subsequent substrate resistance to reaction conditions in the following transformation steps, and adverse effects to the purity of the final probe. As a result, we decided to introduce recently mentioned fluorescent dyes as late as possible in the synthetic sequence. After incorporating the Ala-Phe-Ala chymotrypsin recognizable site surrounded by two polyethylene glycol-based (PEG) spacers, HN6 dye was bound through the sarcosine building block to afford Resin 23. In the next step, Dde protecting group from the lysine moiety placed between caspase-8 and trypsin linkers was removed. Subsequently, fluorescein was attached through the sarcosine scaffold to give Resin 25. Finally, Lys(Mtt) moiety situated between the trypsin and chymotrypsin recognizable sites was deprotected, and Rhodamine B was introduced through the sarcosine segment to provide Resin 27, which was then subjected to chemical cleavage to obtain the final compound 1. In all three cases mentioned, sarcosine served as a secondary amine attachment site for fluorescent dyes, forming corresponding tertiary amides upon binding. That way, fluorescence properties of FL, RhB, and HN6 were enhanced, as their potential ring closure and consequent formation of fluorescence inactive spirolactam scaffolds were disabled. The *tert*-butyloxycarbonyl (Boc) group of lysine from the trypsin cleavage site, as well as sidechain protecting groups of all other building blocks, were efficiently removed during the treatment of the immobilized substrate (Resin 27) with the cleavage cocktail (50% TFA in DCM).

According to LC-MS analysis, still relatively high purity of the immobilized peptide was observed after the binding of HN6 fluorescent dye (ESI – Fig. S1; †Resin 23). However, the last eight reaction steps, including the removal of Dde and Mtt protecting groups, the introduction of Fmoc-Sar-OH in two separate transformations followed by corresponding Fmoc-deprotection, and finally, the introduction of fluorescein and Rhodamine B, resulted in the formation of a highly complex mixture (ESI – Fig. S2–S5†). For purification, a semi-preparative HPLC system equipped with 5 μm 150 × 21.2 mm peptide XB-C18 100 Å column was selected, as it was found superior over 5 μm 100 × 20 mm YMC—Actus Pro C-18 semi-prep column (Kyoto, Japan). The target probe (1) was isolated in the final purity of approximately 72%, as apparent from the LC-MS analysis (ESI – Fig. S7†), performed on a 2.6 μm 150 × 4.6 mm peptide XB-C18 analytical column. The peak of the desired compound was accompanied mainly by the signals of two main impurities that were found to be perfectly covered when LC-MS characterization was carried out on the C-18 XSelect HSS T3 2.5 μm XP (50 × 3.0 mm) column (ESI – Fig. S6†). During the purification process, numerous separation conditions were tested, including various gradients, elution times, mobile phase compositions, usage of different columns, and strict minimization of the injection volumes. As all attempts to obtain the final probe (1) in a purity higher than 72% failed, we decided to evaluate the suitability of the isolated material (1) for the enzyme screening. Similar to a large variety of studies, where biological assays were successfully performed with, *e.g.* crude plant^25,26^ or marine^27^ extracts, we found our substrate (1) suitable for reliable sensing of the studied proteases.

### 3.2. Spectral studies

**Fig. 2:**
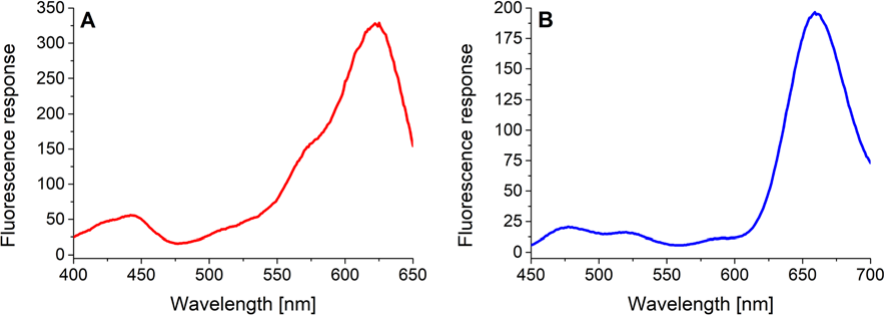
Excitation ((A) *λ*_EMS_ = 665 nm, slit_EXC/EMS_ = 10/5 nm, *c* = 5.0 μM) and emission ((B) *λ*_EXC_ = 425 nm, slit_EXC/EMS_ = 10/10 nm, *c* = 5.3 μM) profiles of the 4-dye probe (1) in Tris–HCl buffer (37 °C).

In the next step, the basic spectral properties of the 4-dye substrate (1) were examined. Excitation and emission profiles were measured in Tris–HCl buffer (pH = 8.0; *T* = 37 °C) at the appropriate emission and excitation wavelengths, respectively. The comprehensive spectral data, including average values and corresponding standard deviations of three independent measurements, are collected in (ESI – Tables S3 and S4†). Fig. 2 presents the fluorescence excitation spectrum (Fig. 2A) measured at the emission wavelength of 665 nm and the fluorescence emission spectrum (Fig. 2B) obtained upon uniform excitation at 425 nm. The complete triple-FRET emission profile of the 4-dye probe (1) in Tris–HCl buffer (Fig. 2B) enabled us to monitor real-time changes in fluorescence responses of all four fluorophores caused by the individual enzyme species.

### 3.3. Solubility and stability testing

Due to its size and complexity, the 4-dye probe (1) was also briefly examined from the solubility in aqueous media point of view. It turned out that at least 0.5 mM stock solution of the target substrate (1) in Tris–HCl buffer (pH = 8.0) could be promptly and efficiently prepared. For comparison, a solution of a hundredfold lower concentration (5 μM) of the 4-dye probe (1) in the same buffer media was utilized in the biological assays. The relatively high solubility of the substrate (1) could be attributed to its structure (Fig. 1), comprising six polyethylene glycol-based (PEG) spacers and two charged fluorophores (Rhodamine B and HN6). Moreover, three terminal carboxyl groups of the peptide backbone are instantly turned into corresponding carboxylate salts in a basic buffer environment, contributing to the solubility of the 4-dye probe (1) in aqueous media. Afterwards, its stability was taken into consideration. The dark blue powder obtained after freeze-drying was dissolved in an appropriate volume of Tris–HCl buffer (pH = 8.0), and shortly after that, LC-MS analysis was performed. The process was repeated a few times within one month. Based on the comparable LC-MS chromatograms, including integrated peaks' areas, we found the 4-dye substrate (1) highly stable for at least 30 days when stored at −80 °C in the form of a solid compound.

### 3.4. Enzyme assays

**Fig. 3:**
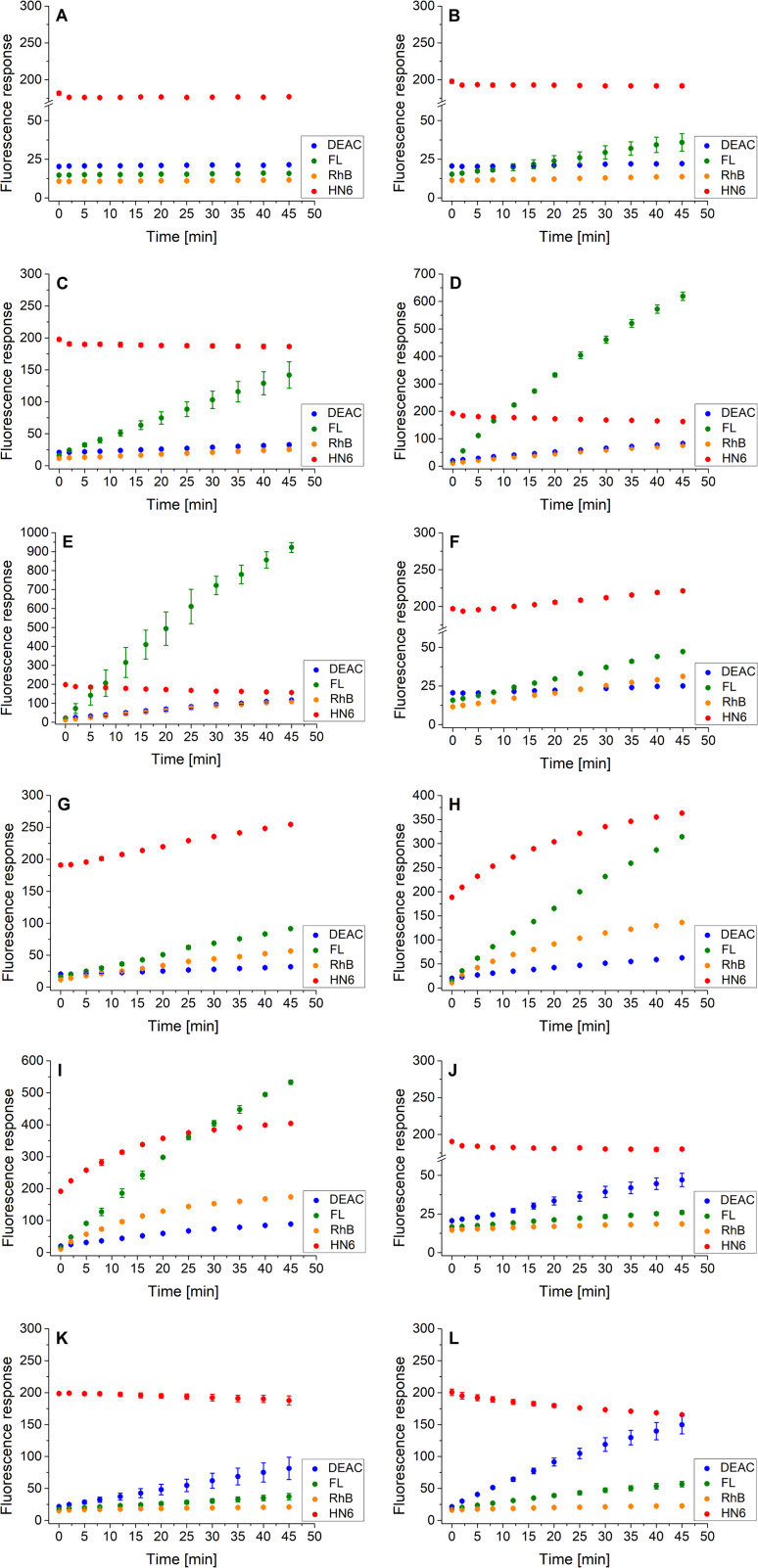
Fluorescence emission responses of the 4-dye probe (1) within the time, in the presence of no enzyme (A), trypsin ((B–E) 0.5 ng mL^−1^, 1 ng mL^−1^, 5 ng mL^−1^, and 10 ng mL^−1^, respectively), chymotrypsin ((F–I) 0.2 μg mL^−1^, 0.5 μg mL^−1^, 2.5 μg mL^−1^, and 5 μg mL^−1^, respectively), and caspase-8 ((J–L) 2 U mL^−1^, 5 U mL^−1^, and 10 U mL^−1^, respectively). The measurements were performed in Tris–HCl buffer (pH = 8.0) at 37 °C. In the case of caspase-8, DTT was added to the buffer. Each measurement was carried out in three independent parallels. The average values and standard deviations were calculated and graphically presented. The numerical data are collected in the (ESI – Tables S5–S16†).

As the three target proteases, trypsin, chymotrypsin, and caspase-8 were considered. While the first two mentioned enzymes are the most common serine proteases, caspase-8 (cysteine-aspartic protease) was chosen due to its selective, reliable cleavage properties and specificity of its recognizable site, which was found to be resistant toward trypsin and chymotrypsin activity. By incorporating selectively cleavable sites for two different classes of proteases into a single substrate, and subsequent unification of experimental conditions, the universality and potential broad applicability of the developed methodology was demonstrated. During the biological testing, the emission responses of all four dyes were monitored throughout the entire period of the experiment. The assay conditions and parameters were established based on our practical observations, theoretical facts, and previous experience.^12,28,29^ To obtain a uniform incubation medium suitable for all three proteases detection, the appropriate conditions for trypsin and chymotrypsin sensing were combined with those suitable for caspase-8 screening. As a result, EDTA, NaCl and glycerol were added to the Tris–HCl buffer solution, and its pH value was set to 8.0. After dissolving the intact 4-dye probe (1) in that way prepared buffer, the emission responses of all four fluorophores were monitored within 45 min of incubation at 37 °C. As can be seen in Fig. 3A, the fluorescence intensities of all four fluorescent dyes remained practically unchanged throughout the experiment period, indicating the substrate's high stability in a buffer solution.

In the next step, the lowest detectable concentrations for all three studied proteases were experimentally determined. While the presence of trypsin and caspase-8 was unambiguously confirmed by the predominant fluorescence rises of fluorescein and coumarin, respectively, chymotrypsin was clearly detectable based on the increase in HN6 emission response. Incubation of the 4-dye substrate (1) with trypsin in its lowest detectable concentration of 0.5 ng mL^−1^ (Fig. 3B) resulted in an insignificant decrease in HN6 intensity and a considerable rise in fluorescein signal. The observed emission trends were further confirmed with the two times (1 ng mL^−1^), ten times (5 ng mL^−1^) and twenty times (10 ng mL^−1^) higher concentrations of trypsin in a sample (Fig. 3C–E). Afterwards, the characteristic pattern for chymotrypsin and its lowest detectable concentration of 0.2 μg mL^−1^ were determined based on the notable rise of fluorescein and HN6 intensities (Fig. 3F). Expectedly, the observed trends were even more apparent when the target substrate was treated with three higher concentrations (0.5 μg mL^−1^, 2.5 μg mL^−1^ and 5.0 μg mL^−1^) of chymotrypsin (Fig. 3G–I). Finally, caspase-8 was taken into consideration. Following the provider's recommendations (Enzo Life Sciences, Inc.), a dl-dithiothreitol (DTT) solution was added to a dissolved substrate in Tris–HCl buffer shortly before adding the lastly-mentioned protease into a sample. Based on a slight decline in the HN6 response and a notable increase of DEAC emission intensity, the presence of caspase-8 in its lowest detectable concentration of 2 U mL^−1^ (Fig. 3J) was clearly recognized. The detected pattern was additionally verified with two higher concentrations (5 U mL^−1^ and 10 U mL^−1^) of caspase-8 in a sample (Fig. 3K and L).

**Fig. 4:**
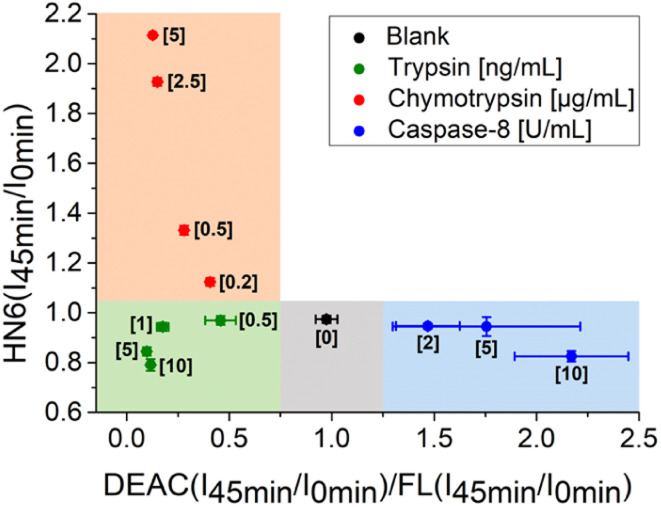
Graphical visualization of individual proteases detection. Ratios of fluorescence emission responses for appropriate fluorophores measured at the times 45 min (*I*_45 min_) and 0 min (*I*_0 min_) were applied. *X* represents DEAC (*I*_45 min_/*I*_0 min_)/FL (*I*_45 min_/*I*_0 min_), while *Y* denotes HN6 (*I*_45 min_/*I*_0 min_). The numerical data are collected in the (ESI – Tables S24 and S25†).

Additionally, the rules for individual protease recognition could be graphically presented, using fluorescence emission ratios of suitable fluorophores measured at particular times. The ratio of fluorescence response change of DEAC and fluorescein within 45 minutes – DEAC (*I*_45 min_/*I*_0 min_)/FL (*I*_45 min_/*I*_0 min_) and HN6 (*I*_45 min_/*I*_0 min_) ratio, were plotted to *X*–*Y* coordinates, respectively. As can be seen in Fig. 4, the absence of enzyme species results in a response in a grey area (0.75 < *X* < 1.25 and *Y* < 1.05). The presence of trypsin in the sample is confirmed by the values in the green sector (*X* < 0.75 and *Y* < 1.05), while the characteristic chymotrypsin range (*X* < 0.75 and *Y* > 1.05) is coloured orange. Finally, the points in the blue field (*X* > 1.25 and *Y* < 1.05) unambiguously identify the presence of caspase-8. The enzymes in concentrations lower than 0.5 ng mL^−1^, 0.2 μg mL^−1^, and 2 U mL^−1^ for trypsin, chymotrypsin, and caspase-8 were found indistinguishable from each other and/or blank value.

**Fig. 5:**
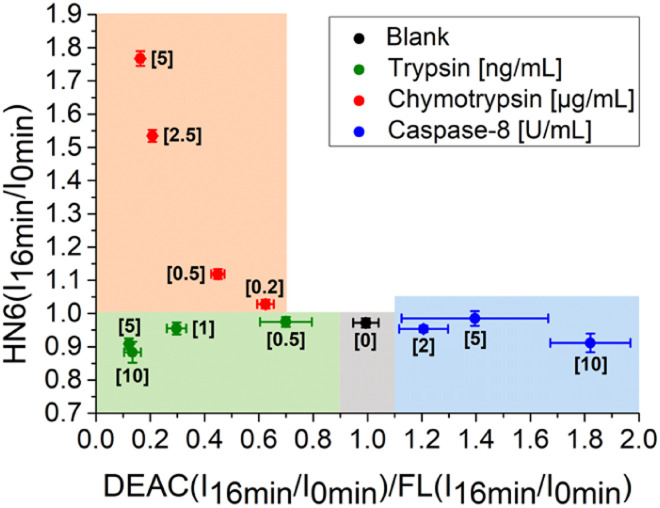
Graphical visualization of individual proteases detection. Ratios of fluorescence emission responses for appropriate fluorophores measured at the times 16 min (*I*_16 min_) and 0 min (*I*_0 min_) were applied. *X* represents DEAC (*I*_16 min_/*I*_0 min_)/FL (*I*_16 min_/*I*_0 min_), while *Y* denotes HN6 (*I*_16 min_/*I*_0 min_). The numerical data are collected in the (ESI – Tables S26 and S27†).

In the next step, we focused on optimizing of the time suitable for biological assays. Using a slightly modified, recently presented model (Fig. 4) for the detection of individual proteases, we concluded that the tested enzymes were clearly distinguishable in the defined concentration ranges after only 16 minutes of incubation with the 4-dye substrate (Fig. 5). While maintaining the colours of the individual ranges, the new limits were set as follows: 0.90 < *X* < 1.10 and *Y* < 1.00 for blank, *X* < 0.90 and *Y* < 1.00 for trypsin, *X* < 0.70 and *Y* > 1.00 for chymotrypsin, and *X* > 1.10 and *Y* < 1.05 for caspase-8.

Following the successful development of the methodology for the separate detection of individual proteases in a sample, we decided to investigate and evaluate the possibilities of simultaneous detection of multiple enzymes. Whereas caspase-8 and trypsin were shown to cleave only their own recognizable sites (ESI – Fig. S9 and S11†), both chymotrypsin and trypsin linkers were cleaved in the presence of chymotrypsin (ESI – Fig. S10†). Consequently, some potential ambiguities and drawbacks were expected from the perspective of nonspecific cleavage of the 4-dye substrate 1 by chymotrypsin. The experiments of the two-enzyme detection were performed with all three theoretically possible pairs of the considered proteases in their low concentrations. For this purpose, the single-enzyme assay described above was slightly modified and dl-dithiothreitol (DTT) was added to the sample after 20 minutes of incubation, which resulted in the immediate deactivation of the serine proteases (trypsin, chymotrypsin), whereas the cysteine-aspartic protease (caspase-8) remained active.

**Fig. 6:**
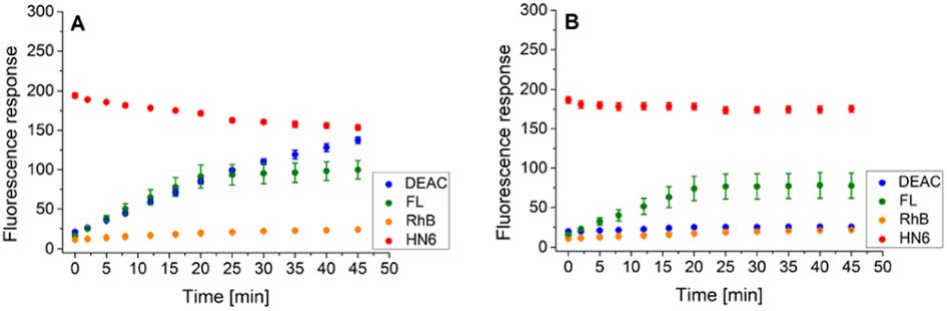
Fluorescence emission responses of the 4-dye probe (1) within the time, in the presence of caspase-8/trypsin pair (A), and solely trypsin (B). The measurements were performed in Tris–HCl buffer (pH = 8.0), at 37 °C. After 20 min of incubation, DTT was added. Each measurement was carried out in three independent parallels. The average values and standard deviations were calculated and graphically presented. The numerical data are collected in the (ESI – Tables S17 and S18†).

Following this procedure, we first examined the cleavage of the caspase-8/trypsin enzyme pair (Fig. 6A) and compared it with the activity of trypsin itself (Fig. 6B). While the trends for FL, RhB, and HN6 were comparable in both cases, the main difference was observed in the DEAC fluorescence response. In the exclusive presence of trypsin (1 ng mL^−1^) (Fig. 6B), the emission intensity of DEAC remained low and almost unchanged throughout the experimental period. In contrast, in the simultaneous presence of caspase-8 (2 U mL^−1^) and trypsin (1 ng mL^−1^), a significant and gradual increase in the coumarin fluorescence signal was observed throughout the incubation period (Fig. 6A), allowing a clear distinction between the two scenarios.

**Fig. 7:**
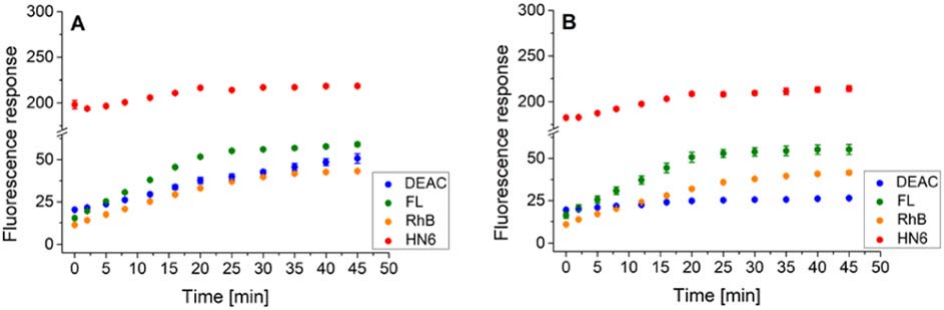
Fluorescence emission responses of the 4-dye probe (1) within the time, in the presence of caspase-8/chymotrypsin pair (A), and solely chymotrypsin (B). The measurements were performed in Tris–HCl buffer (pH = 8.0), at 37 °C. After 20 min of incubation, DTT was added. Each measurement was carried out in three independent parallels. The average values and standard deviations were calculated and graphically presented. The numerical data are collected in the (ESI – Tables S19 and S20†).

Similarly, the enzyme activities of the caspase-8/chymotrypsin pair (Fig. 7A) were considered, and the observed trends in fluorophore emission responses were compared with those caused by chymotrypsin cleavage alone (Fig. 7B). In this case, the increase in DEAC fluorescence intensity was also notable, although less significant than for the caspase-8/trypsin pair. Nevertheless, the presence of chymotrypsin alone (0.5 μg mL^−1^) was still clearly distinguishable from the mixture of caspase-8 (2 U mL^−1^) and chymotrypsin (0.5 μg mL^−1^). While for the former, the coumarin emission response reached a plateau after addition of dl-dithiothreitol (Fig. 7B), in case of the latter, a gradual increase in DEAC signal was observed throughout the 45 minutes of the assay (Fig. 7A).

**Fig. 8:**
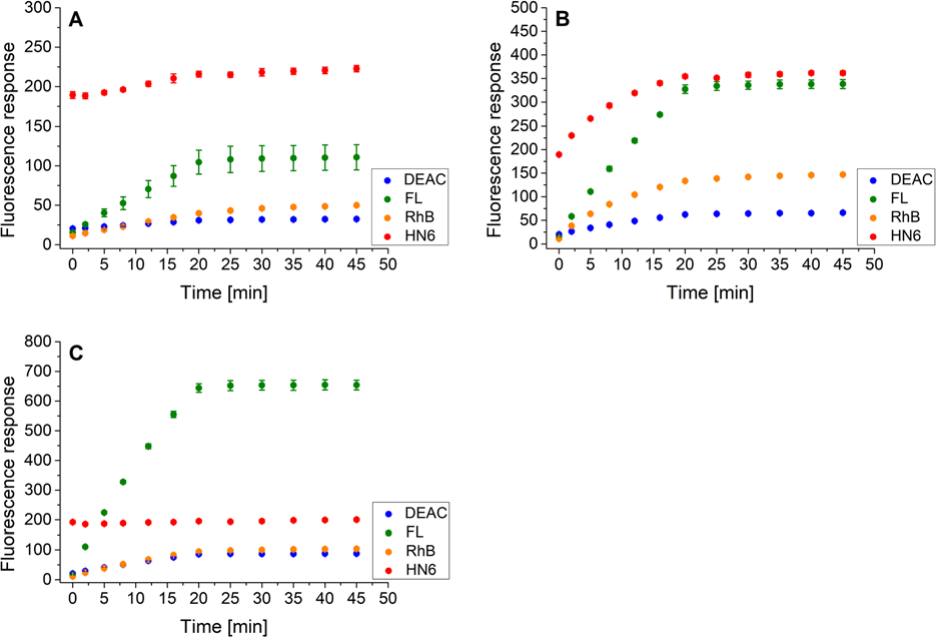
Fluorescence emission responses of the 4-dye probe (1) within the time, in the co-presence of trypsin (low: 1 ng mL^−1^, high: 10 ng mL^−1^) and chymotrypsin (low: 0.5 μg mL^−1^, high: 5.0 μg mL^−1^): low_TRYP_–low_CHYM_ (A), low_TRYP_–high_CHYM_ (B), and high_TRYP_–low_CHYM_ (C). The measurements were performed in Tris–HCl buffer (pH = 8.0), at 37 °C. After 20 min of incubation, DTT was added. Each measurement was carried out in three independent parallels. The average values and standard deviations were calculated and graphically presented. The numerical data are collected in the (ESI – Tables S21–S23†).

Finally, the same methodology, including the addition of DTT after 20 minutes of the experiment, was applied to the trypsin/chymotrypsin pair. When low concentrations of the two proteases were administered, the presence of chymotrypsin was clearly evident due to the marked increase in fluorescein and HN6 intensities during the first 20 minutes of the experiment. On the other hand, trypsin was not readily detectable because it was masked by chymotrypsin (Fig. 8A). As expected, a similar situation was observed when a higher amount of chymotrypsin was used while the trypsin concentration was kept low (Fig. 8B). In contrast, when a high trypsin concentration and a low chymotrypsin concentration were used, the virtually unchanged HN6 emission intensity and the sharp increase in fluorescein response within the first 20 minutes of an assay indicated the presence of trypsin, whereas the detection of chymotrypsin in a mixture remained unclear (Fig. 8C). Thus, we had to conclude that reliable simultaneous detection of trypsin and chymotrypsin is not feasible, mainly due to the nonselective cleavage activity of chymotrypsin and the mutual masking of the two proteases in different concentration combinations. Therefore, we did not investigate synchronous recognition of all three proteases in a mixture.

## 4. Conclusions

In summary, a fluorescent multipurpose triple-FRET 4-dye peptide probe 1 was synthesized using a multistep Fmoc-based solid-phase approach and successfully used for the detection of trypsin, chymotrypsin, and caspase-8 in a single-enzyme mode. Subsequently, screening of individual protease pairs was considered, but proved only partially feasible, mainly due to the nonselective cleavage activity of chymotrypsin. A number of similar fluorescent substrates could be obtained inexpensively by simple modification of individual recognizable sites. Consequently, the methodology presented here could be elegantly generalized to any other protease trio, as disruption of specific linkers should always result in the same fluorophores' emission patterns, completely independent of the cleaving agents. The robustness and tolerance to impurities of the developed method, as well as the use of inexpensive instrumentation and uncomplicated analytical techniques, could be readily applicable to simple, yet reliable versatile sensors fabrication.

## Author contributions

Conceptualization and methodology: D. M. and J. H.; investigation: D. M.; writing—original draft preparation, reviewing and editing: D. M. and J. H.; funding acquisition: J. H. All authors have read and agreed to the published version of the manuscript.

## Conflicts of interest

There are no conflicts to declare.

## Supplementary Material

RA-012-D2RA05125G-s001
