# Supplementary material for: Triple-FRET multi-purpose fluorescent probe for three-protease detection
Source: RSC Adv. 2022 Oct 10;12(44):28780–7. doi: 10.1039/d2ra05125g (PMC9549473; doi:10.1039/d2ra05125g)
Supplement: RA-012-D2RA05125G-s001 [file RA-012-D2RA05125G-s001.pdf]

Electronic Supplementary Material (ESI) for RSC Advances.
This journal is © The Royal Society of Chemistry 2022

Supplementary Information

Triple-FRET multi-purpose fluorescent probe for three-protease detection

David Milićević [a] and Jan Hlaváč*[a]

[a]Department of Organic Chemistry, Faculty of Science, Palacký University Olomouc, 17. Listopadu 12, 771 46 Olomouc, Czech Republic. *E-mail: jan.hlavac@upol.cz

Table of Contents

1. Synthesis 2
2. Photophysical properties and spectral data 11
3. Biological assays 13

1. Synthesis

Scheme S1: Synthesis of the 4-dye probe on Polystyrene Wang resin.

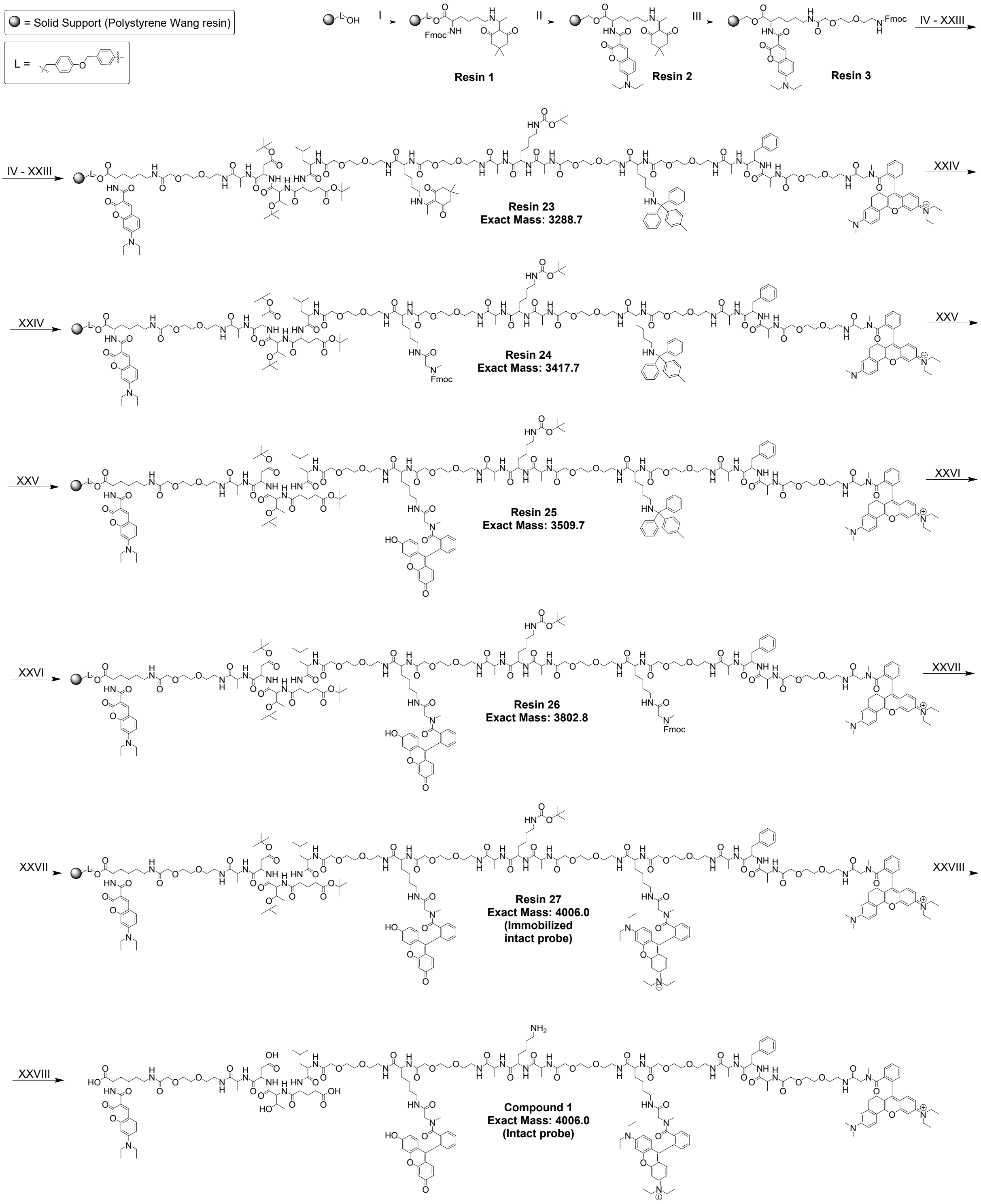

I. Fmoc-Lys(Dde)-OH, HOBt, DMAP, DIC, DMF:DCM 1:1, rt, 16h; **II.** a.) 50% piperidine in DMF, rt, 30 min; b.) DEAC, HOBt, DMAP, DIC, DMF:DCM:DMSO 1:2:2, rt, 16h; **III**. a.) $HONH_2$·HCl, imidazole, NMP:DCM 3:2, rt, 3h; b.) PEG, HOBt, DIC, DMF:DCM 1:1, rt, 2h; **IV.** a.) 50% piperidine in DMF, rt, 30 min; b.) Fmoc-Ala-OH, HOBt, DIC, DMF:DCM 1:1, rt, 2h; **V.** a.) 50% piperidine in DMF, rt, 30 min; b.) Fmoc-Asp(OtBu)-OH, HOBt, DIC, DMF:DCM 1:1, rt, 2h; **VI.** a.) 50% piperidine in DMF, rt, 30 min; b.) Fmoc-Thr(tBu)-OH, HOBt, DIC, DMF:DCM 1:1, rt, 2h; **VII.** a.) 50% piperidine in DMF, rt, 30 min; b.) Fmoc-Glu(OtBu)-OH, HOBt, DIC, DMF:DCM 1:1, rt, 2h; **VIII.** a.) 50% piperidine in DMF, rt, 30 min; b.) Fmoc-Leu-OH, HOBt, DIC, DMF:DCM 1:1, rt, 2h; **IX.** a.) 50% piperidine in DMF, rt, 30 min; b.) PEG, HOBt, DIC, DMF:DCM 1:1, rt, 2h; **X.** a.) 50% piperidine in DMF, rt, 30 min; b.) Fmoc-Lys(Dde)-OH, HOBt, DIC, DMF:DCM 1:1, rt, 2h; **XI.** a.) 50% piperidine in DMF, rt, 30 min; b.) PEG, HOBt, DIC,

DMF:DCM 1:1, rt, 3h; **XII.** a.) 50% piperidine in DMF, rt, 30 min; b.) Fmoc-Ala-OH, HOBt, DIC, DMF:DCM 1:1, rt, 3h; **XIII.** a.) 50% piperidine in DMF, rt, 30 min; b.) Fmoc-Lys(Boc)-OH, HOBt, DIC, DMF:DCM 1:1, rt, 3h; **XIV.** a.) 50% piperidine in DMF, rt, 30 min; b.) Fmoc-Ala-OH, HOBt, DIC, DMF:DCM 1:1, rt, 3h; **XV.** a.) 50% piperidine in DMF, rt, 30 min; b.) PEG, HOBt, DIC, DMF:DCM 1:1, rt, 3h; **XVI.** a.) 50% piperidine in DMF, rt, 30 min; b.) Fmoc-Lys(Mtt)-OH, HOBt, DIC, DMF:DCM 1:1, rt, 3h; **XVII.** a.) 50% piperidine in DMF, rt, 45 min; b.) PEG, HOBt, DIC, DMF:DCM 1:1, rt, 3h; **XVIII.** a.) 50% piperidine in DMF, rt, 45 min; b.) Fmoc-Ala-OH, HOBt, DIC, DMF:DCM 1:1, rt, 3h; **XIX.** a.) 50% piperidine in DMF, rt, 45 min; b.) Fmoc-Phe-OH, HOBt, DIC, DMF:DCM 1:1, rt, 3h; **XX.** a.) 50% piperidine in DMF, rt, 45 min; b.) Fmoc-Ala-OH, HOBt, DIC, DMF:DCM 1:1, rt, 3h; **XXI.** a.) 50% piperidine in DMF, rt, 45 min; b.) PEG, HOBt, DIC, DMF:DCM 1:1, rt, 3h; **XXII.** a.) 50% piperidine in DMF, rt, 45 min; b.) Fmoc-Sar-OH, HOBt, DIC, DMF:DCM 1:1, rt, 3h; **XXIII.** a.) 50% piperidine in DMF, rt, 45 min; b.) HN6, HOBt, DMAP, DIC, DMF:DCM:DMSO 1:2:2, rt, 16h; **XXIV.** a.) $HONH_2 \cdot HCl$, imidazole, NMP:DCM 3:2, rt, 2.5h; b.) Fmoc-Sar-OH, HOBt, DIC, DMF:DCM 1:1, rt, 3h; **XXV.** a.) 50% piperidine in DMF, rt, 45 min; b.) FL, HOBt, DMAP, DIC, DMF:DCM:DMSO 1:2:2, rt, 16h; **XXVI.** a.) DCE, TES, HFIP, TFE, 60 °C, 5h; b.) Fmoc-Sar-OH, HOBt, DIC, DMF:DCM 1:1, rt, 3h; **XXVII.** a.) 50% piperidine in DMF, rt, 45 min; b.) RhB, HOBt, DMAP, DIC, DMF:DCM:DMSO 1:2:2, rt, 16h; **XXVIII.** 50% TFA in DCM, rt, 60 min.

Molecular weights of cleaved peptides are reported. Tert-butyl (tBu), tert-butyloxycarbonyl (Boc) and 4-methyltrityl (Mtt) protecting groups are removed during the chemical cleavage with 50% TFA in DCM.

Resin 1

A resin was washed with dichloromethane (5x), and subsequently reacted with Fmoc-Lys(Dde)-OH (2.0 mmol), HOBt (2.0 mmol), DMAP (0.5 mmol), and DIC (2.0 mmol) in DMF (5 mL) and DCM (5 mL). The reaction mixture was shaken for 16 hours at lab temperature. Afterwards, a solid support was washed with DMF (10x) and DCM (10x).

Resin 2

Resin 1 was subjected to 50% piperidine in DMF for 30 min, and subsequently washed with DMF (10x) and DCM (10x).
Then, the resin was reacted with DEAC (2.0 mmol), HOBt (2.0 mmol), DMAP (2.0 mmol), and DIC (2.0 mmol) in DMF (2 mL), DCM (4 mL), and DMSO (4 mL). The reaction mixture was shaken for 16 hours at lab temperature. Afterwards, a solid support was washed with DMSO (5X), DMF (10x) and DCM (10x).

Resin 3

To Resin 2, $HONH_2 \cdot HCl$ (9.0 mmol) and imidazole (6.7 mmol) in NMP (6 mL) and DCM (4 mL) were added, and obtained heterogeneous mixture was shaken for 3 hours at lab temperature. Afterwards, a solid support was washed with NMP (5X), DMF (10x) and DCM (10x).
Then, the resin was reacted with PEG (2.0 mmol), HOBt (2.0 mmol), and DIC (2.0 mmol) in DMF (5 mL) and DCM (5 mL). The reaction mixture was shaken for 2 hours at lab temperature. Afterwards, a solid support was washed with DMF (10x) and DCM (10x).

Resin 4–22

An appropriate resin was subjected to 50% piperidine in DMF for 30–45 min, and subsequently washed with DMF (10x) and DCM (10x).
Then, a solid support was reacted with a suitable amino acid or PEG spacer (2.0 mmol), HOBt (2.0 mmol), and DIC (2.0 mmol) in DMF (5 mL) and DCM (5 mL). A reaction mixture was shaken for 2–3 hours at lab temperature. Afterwards, a solid support was washed with DMF (10x) and DCM (10x).

Resin 23

Resin 22 was subjected to 50% piperidine in DMF for 45 min, and subsequently washed with DMF (10x) and DCM (10x). Then, the resin was reacted with HN6 (2.0 mmol), HOBt (2.0 mmol), DMAP (2.0 mmol), and DIC (2.0 mmol) in DMF (2 mL), DCM (4 mL), and DMSO (4 mL). The reaction mixture was shaken for 16 hours at lab temperature. Afterwards, a solid support was washed with DMSO (5X), DMF (10x) and DCM (10x).

Figure S1: LC-MS analysis of chemically cleaved peptide from Resin 23 (Rt = 2.76 min).

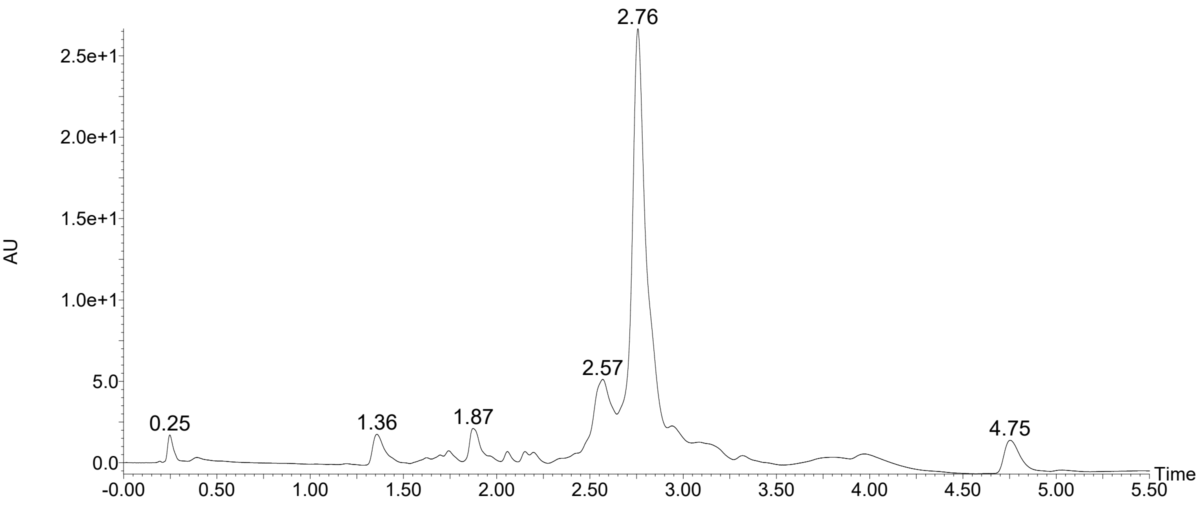

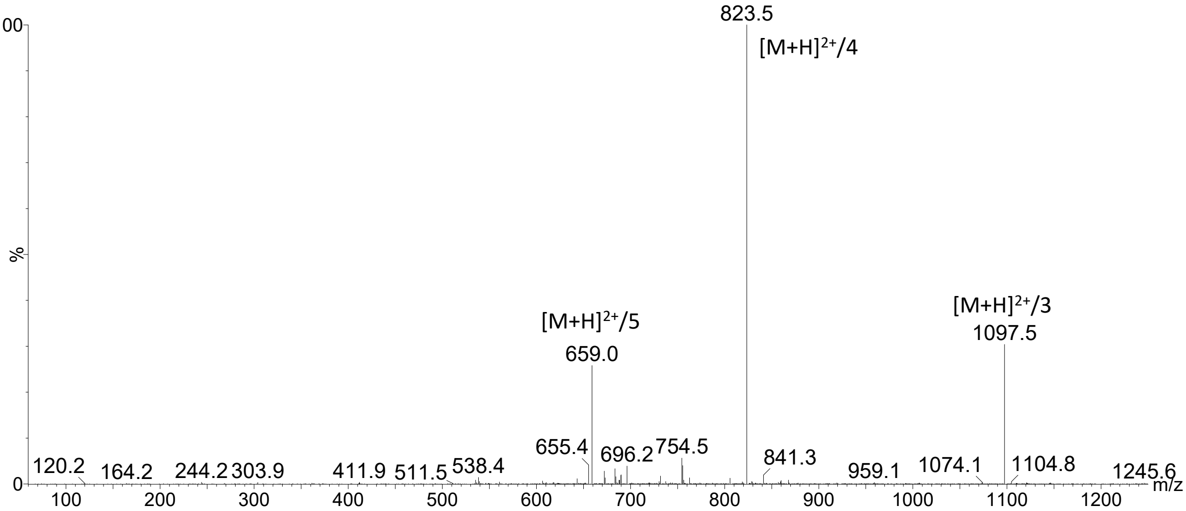

Method: Ammonium acetate (10 mM) in ultrapure water and acetonitrile (gradient 20–80% during the first 4.5 min). LC-MS column: (50 x 3.0 mm XSelect HSS T3 2.5 µm XP C18, Waters, Borehamwood, UK).

Resin 24

To Resin 23, $HONH_2 \cdot HCl$ (9.0 mmol) and imidazole (6.7 mmol) in NMP (6 mL) and DCM (4 mL) were added, and obtained heterogeneous mixture was shaken for 2.5 hours at lab temperature. Afterwards, a solid support was washed with NMP (5X), DMF (10x) and DCM (10x).
Then, the resin was reacted with Fmoc-Sar-OH (2.0 mmol), HOBt (2.0 mmol), and DIC (2.0 mmol) in DMF (5 mL) and DCM (5 mL). The reaction mixture was shaken for 3 hours at lab temperature. Afterwards, a solid support was washed with DMF (10x) and DCM (10x).

Figure S2: LC-MS analysis of chemically cleaved peptide from Resin 24 (Rt = 3.05 min).

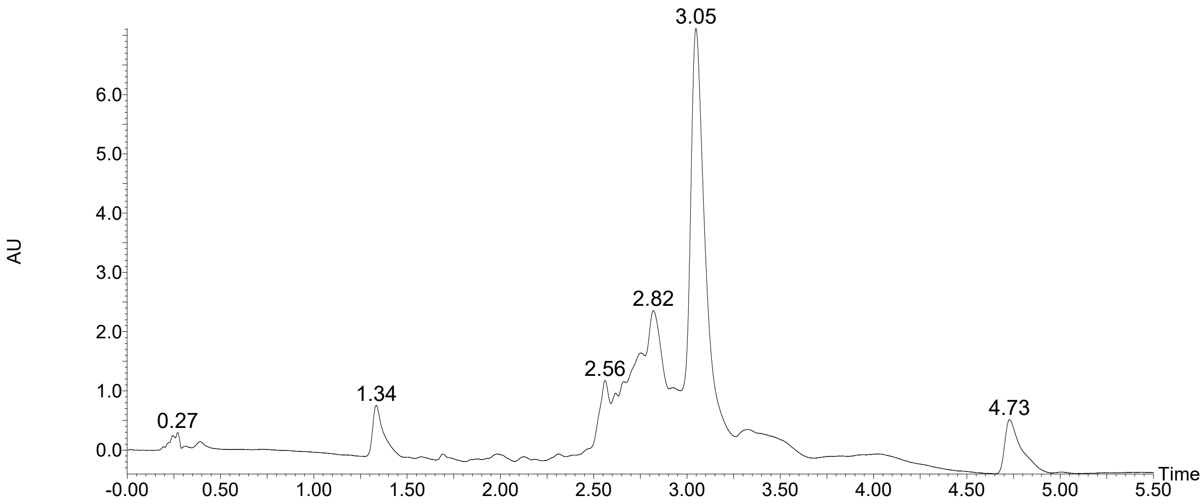

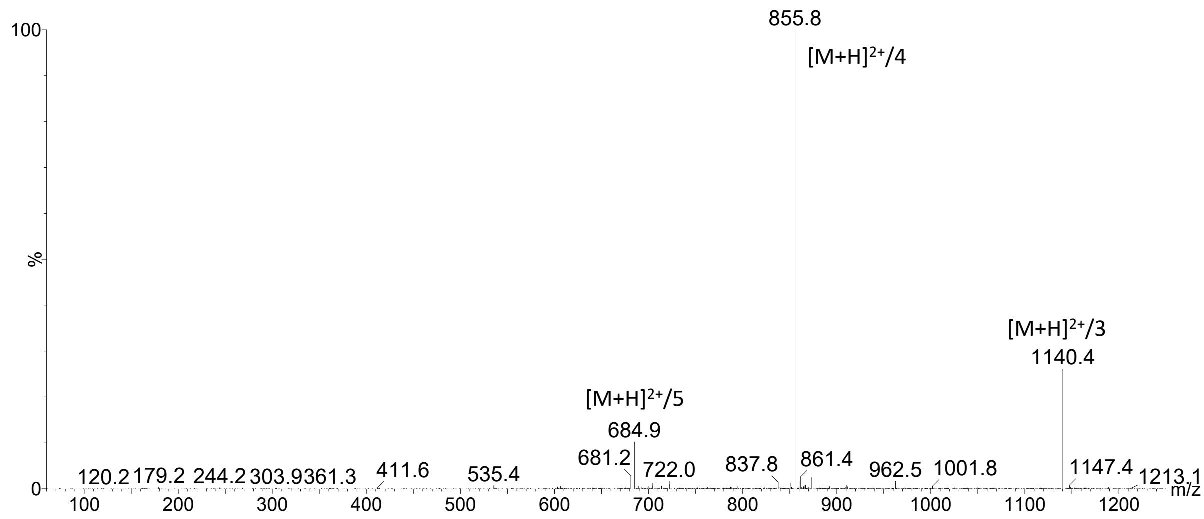

Method: Ammonium acetate (10 mM) in ultrapure water and acetonitrile (gradient 20–80% during the first 4.5 min).
LC-MS column: (50 x 3.0 mm XSelect HSS T3 2.5 µm XP C18, Waters, Borehamwood, UK).

<u>Resin 25</u>

Resin 24 was subjected to 50% piperidine in DMF for 45 min, and subsequently washed with DMF (10x) and DCM (10x). Then, the resin was reacted with FL (2.0 mmol), HOBt (2.0 mmol), DMAP (2.0 mmol), and DIC (2.0 mmol) in DMF (2 mL), DCM (4 mL), and DMSO (4 mL). The reaction mixture was shaken for 16 hours at lab temperature. Afterwards, a solid support was washed with DMSO (5X), DMF (10x) and DCM (10x).

Figure S3: LC-MS analysis of chemically cleaved peptide from Resin 25 (Rt = 2.54 min).

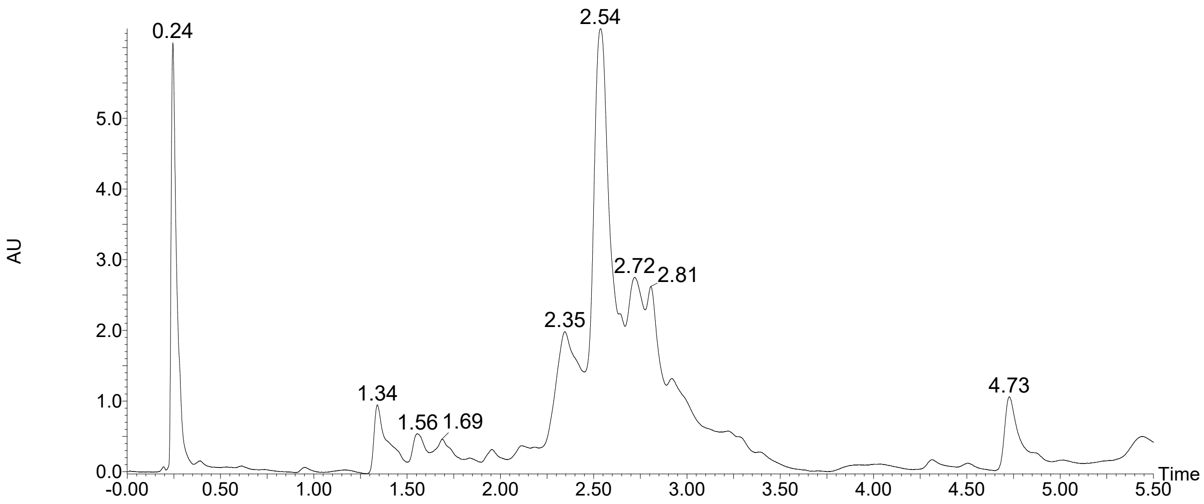

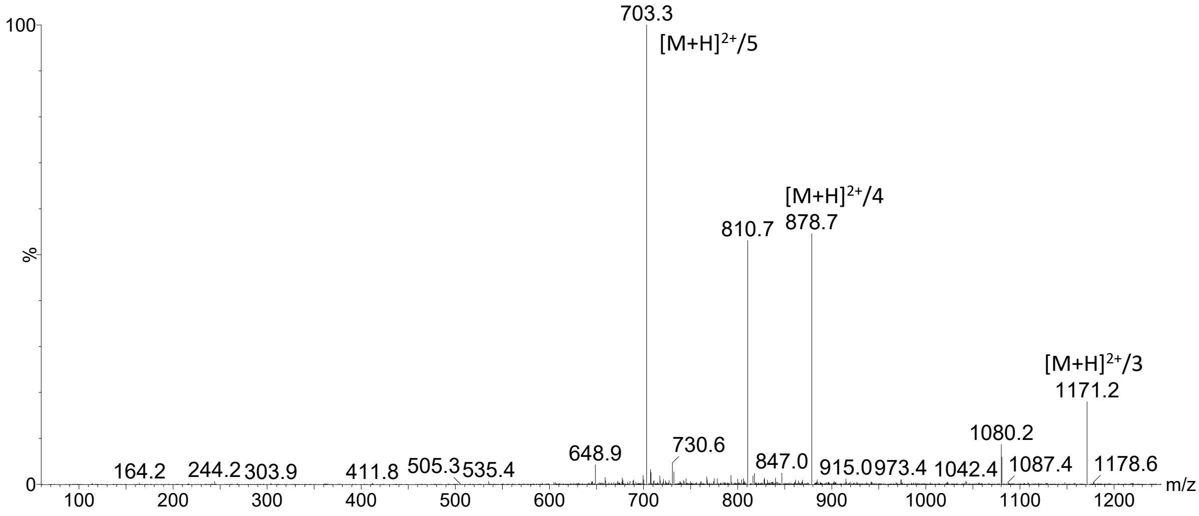

Method: Ammonium acetate (10 mM) in ultrapure water and acetonitrile (gradient 20–80% during the first 4.5 min). LC-MS column: (50 x 3.0 mm XSelect HSS T3 2.5 µm XP C18, Waters, Borehamwood, UK).

Resin 26

To Resin 25 (300 mg), 1,2-dichloroethane (9.75 mL), triethylsilane (3.0 mL), hexafluoroisopropanol (1.5 mL) and trifluoroethanol (0.75 mL) were added. This way obtained heterogeneous mixture was at 60 °C shaken for the time period of 5 hours. Afterwards, a solid support was washed with DCM (20x).
Then, the resin was reacted with Fmoc-Sar-OH (2.0 mmol), HOBt (2.0 mmol), and DIC (2.0 mmol) in DMF (5 mL) and DCM (5 mL). The reaction mixture was shaken for 3 hours at lab temperature. Afterwards, a solid support was washed with DMF (10x) and DCM (10x).

Figure S4: LC-MS analysis of chemically cleaved peptide from Resin 26 (Rt = 2.97 min).

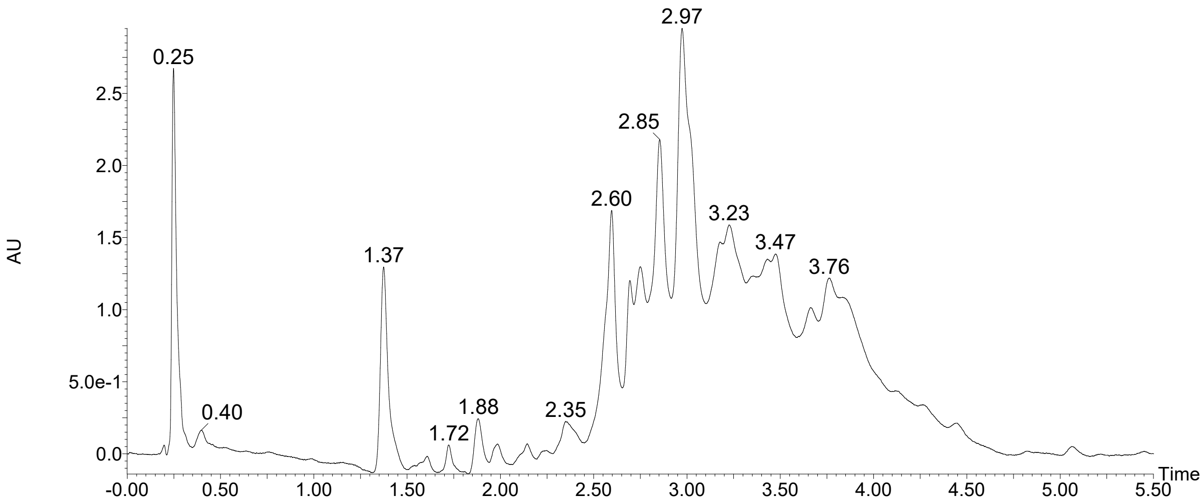

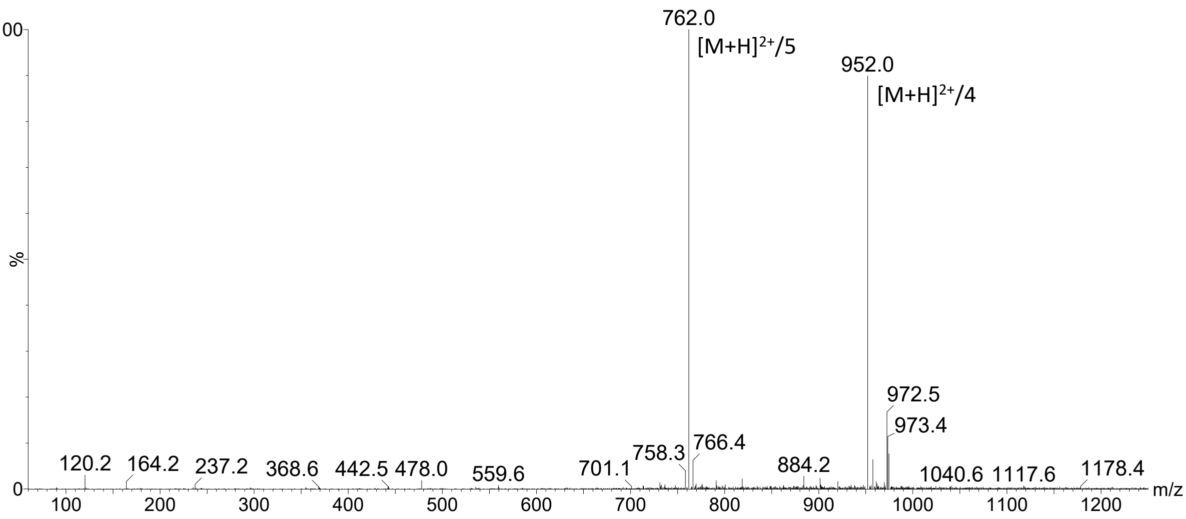

Method: Ammonium acetate (10 mM) in ultrapure water and acetonitrile (gradient 20–80% during the first 4.5 min).
LC-MS column: (50 x 3.0 mm XSelect HSS T3 2.5 µm XP C18, Waters, Borehamwood, UK).

Resin 27

Resin 26 was subjected to 50% piperidine in DMF for 45 min, and subsequently washed with DMF (10x) and DCM (10x). Then, the resin was reacted with RhB (2.0 mmol), HOBt (2.0 mmol), DMAP (2.0 mmol), and DIC (2.0 mmol) in DMF (2 mL), DCM (4 mL), and DMSO (4 mL). The reaction mixture was shaken for 16 hours at lab temperature. Afterwards, a solid support was washed with DMSO (5X), DMF (10x) and DCM (10x).

Figure S5: LC-MS analysis of chemically cleaved peptide from Resin 27 (Rt = 3.17 min).

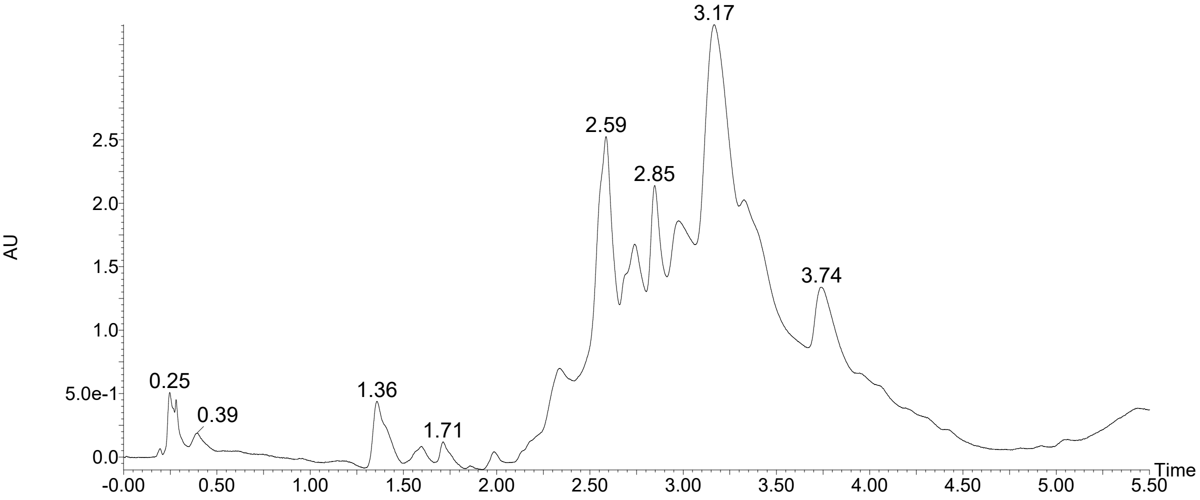

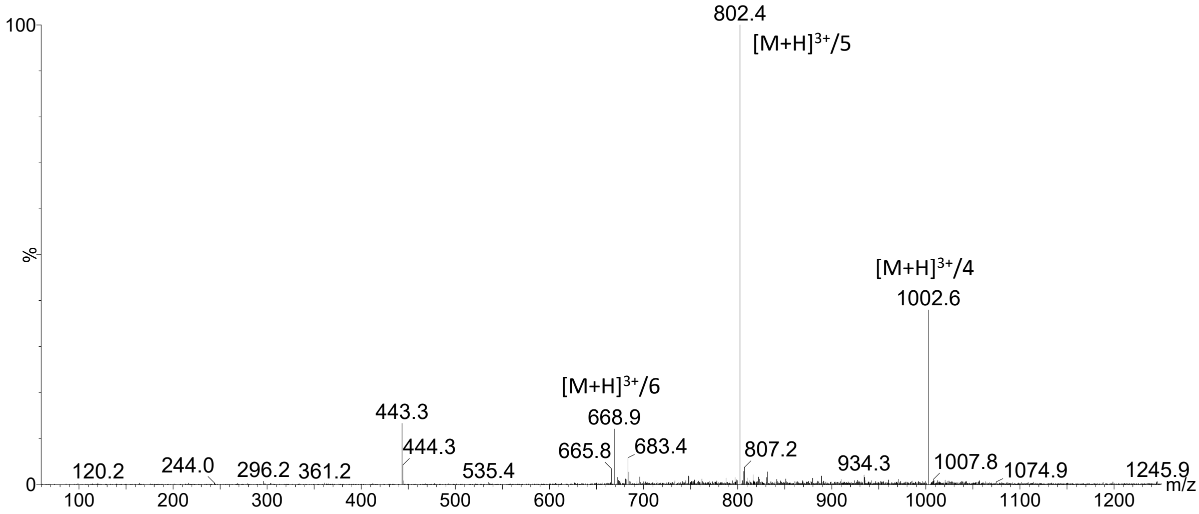

Method: Ammonium acetate (10 mM) in ultrapure water and acetonitrile (gradient 20–80% during the first 4.5 min). LC-MS column: (50 x 3.0 mm XSelect HSS T3 2.5 µm XP C18, Waters, Borehamwood, UK).

Figure S6: LC-MS analysis of isolated intact 4-dye probe after freeze-drying (Rt = 3.15 min).

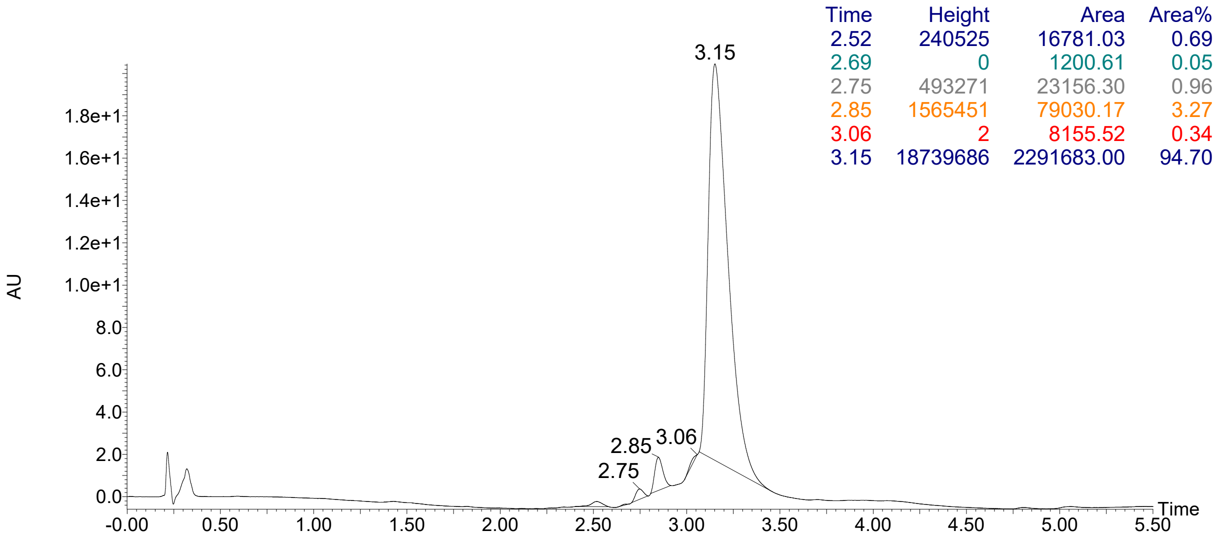

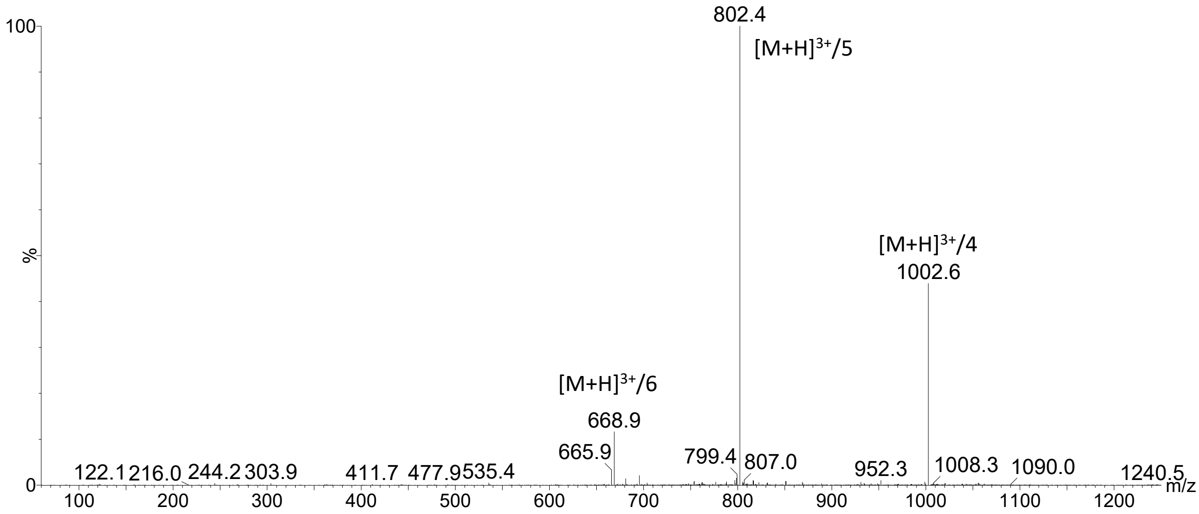

Method: Ammonium acetate (10 mM) in ultrapure water and acetonitrile (gradient 20–80% during the first 4.5 min).
LC-MS column: (50 x 3.0 mm XSelect HSS T3 2.5 µm XP C18, Waters, Borehamwood, UK).

Figure S7: LC-MS analysis of isolated intact 4-dye probe (Rt = 18.94 min).

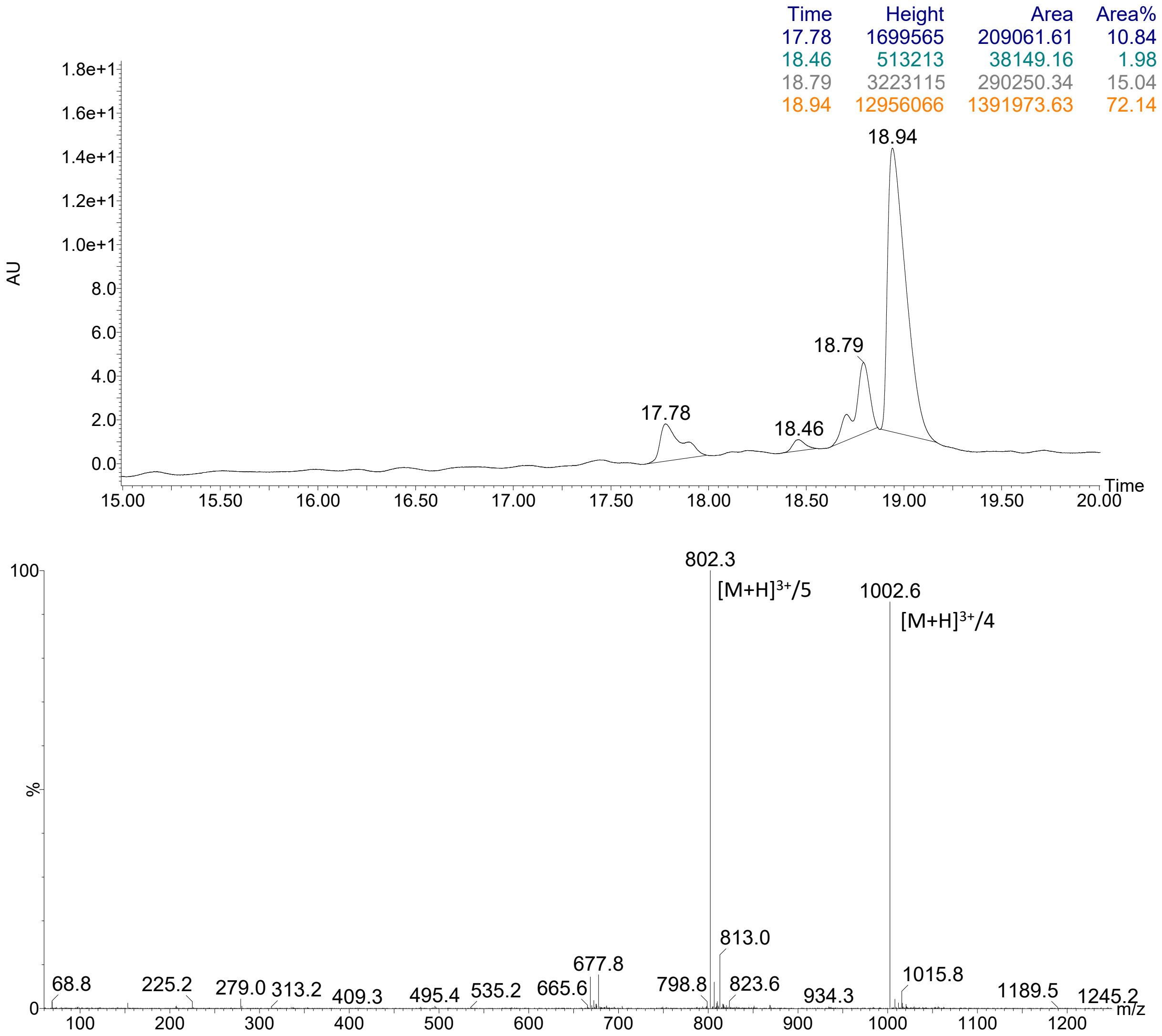

Method: Formic acid (0.1%) in ultrapure water (*V*/*V*) and acetonitrile (gradient 20–52% during the first 20 min).
LC-MS column: (150 x 4.6 mm bioZen 2.6 µm peptide XB-C18, Phenomenex, USA).

Figure S8: HRMS analysis of isolated intact 4-dye probe.

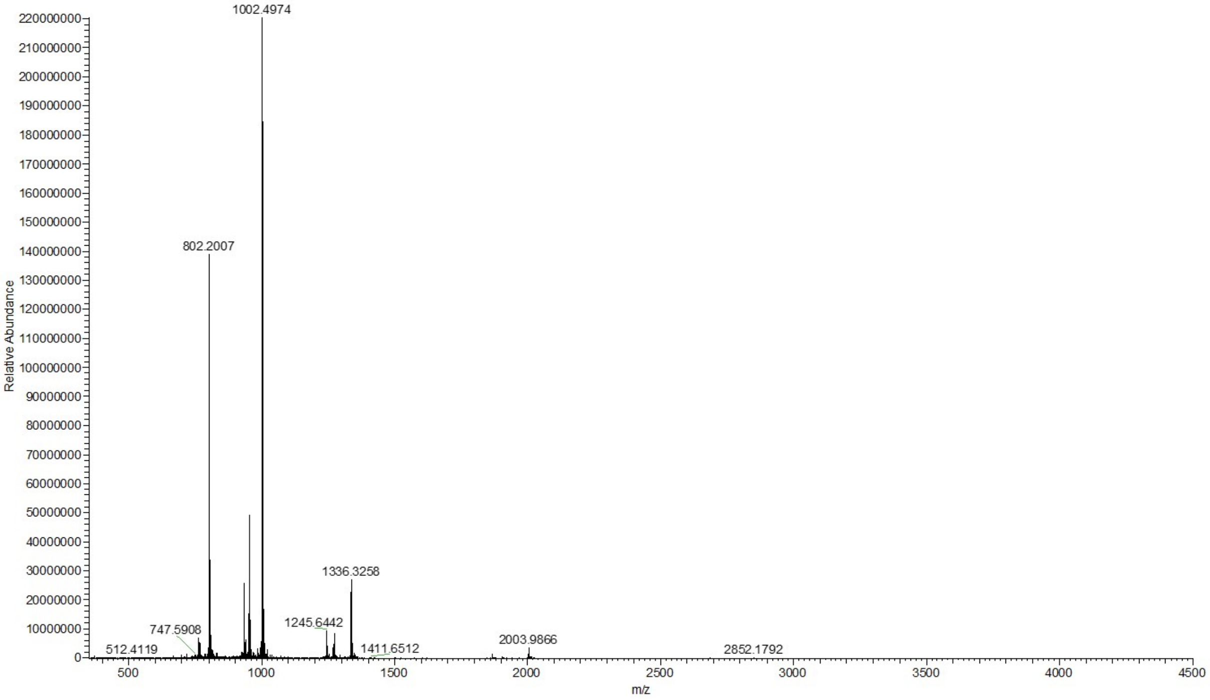

HRMS (ESI):
m/z calcd $C_{204}H_{276}N_{32}O_{52}{}^{2+}$ for $[M+2H]^{2+}/2$ = 2004.0036, found $[M+2H]^{2+}/2$ = 2003.9866;
m/z calcd $C_{204}H_{276}N_{32}O_{52}{}^{2+}$ for $[M+3H]^{3+}/3$ = 1336.3381, found $[M+3H]^{3+}/3$ = 1336.3258;
m/z calcd $C_{204}H_{276}N_{32}O_{52}{}^{2+}$ for $[M+4H]^{4+}/4$ = 1002.5054, found $[M+4H]^{4+}/4$ = 1002.4974;
m/z calcd $C_{204}H_{276}N_{32}O_{52}{}^{2+}$ for $[M+5H]^{5+}/5$ = 802.2058, found $[M+5H]^{5+}/5$ = 802.2007.

2. Photophysical properties and spectral data

Table S1: Quantum yields of individual fluorophores of the intact 4-dye substrate.

Coumarin	Fluorescein	Rhodamine B	HN6	Reference
<0.01	<0.01	<0.01	0.14	Fluorescein (0.1 M NaOH)
0.05	0.03	0.01	0.69	Rhodamine 6G (water)
0.03	0.02	<0.01	0.43	Rhodamine B (water)

Table S2: FRET efficiency between individual FRET couples and total FRET efficiency of the intact 4-dye substrate.

DEAC → FL	FL → RhB	RhB → HN6	Total
0.67	0.69	0.80	0.37

Table S3: Fluorescence emission intensities at corresponding excitation wavelengths.

Emission [nm]	$Slit_{EXC/EMS}$: 10/10 Average	$Slit_{EXC/EMS}$: 10/10 St. Dev.	$Slit_{EXC/EMS}$: 10/5 Average	$Slit_{EXC/EMS}$: 10/5 St. Dev.	Excitation [nm]
480	20.11	0.31	5.28	0.12	433
522	18.21	0.46	4.56	0.07	433
595	11.72	0.38	2.94	0.10	433
665	203.77	2.17	51.53	0.51	433
480	/	/	/	/	500
522	63.02	2.18	16.35	0.53	500
595	24.53	0.76	6.21	0.32	500
665	110.28	0.74	28.18	0.16	500
480	/	/	/	/	565
522	/	/	/	/	565
595	129.27	4.41	32.72	0.89	565
665	520.72	4.11	131.22	1.56	565
480	/	/	/	/	622
522	/	/	/	/	622
595	/	/	/	/	622
665	1000+	-	330.15	4.00	622

Preparation: 5 µM 4-dye probe in Tris-HCl buffer (pH=8.0) (1 mL); Incubation: T=37 °C. All measurements were performed in three parallels. The corresponding average values and standard deviations are reported.

Table S4: Fluorescence excitation intensities at corresponding emission wavelengths.

Excitation [nm]	$Slit_{EXC/EMS}$: 10/10 Average	$Slit_{EXC/EMS}$: 10/10 St. Dev.	$Slit_{EXC/EMS}$: 10/5 Average	$Slit_{EXC/EMS}$: 10/5 St. Dev.	Emission [nm]
433	20.20	0.60	5.22	0.16	480
500	/	/	/	/	480
565	/	/	/	/	480
622	/	/	/	/	480
433	18.02	0.65	4.60	0.15	522
500	62.40	2.27	16.06	0.57	522
565	/	/	/	/	522
622	/	/	/	/	522
433	11.72	0.22	2.95	0.09	595
500	24.16	0.77	6.11	0.11	595
565	126.36	3.49	32.00	1.15	595
622	/	/	/	/	595
433	203.98	0.91	51.25	0.27	665
500	110.25	0.56	27.62	0.28	665
565	520.15	3.78	132.66	0.69	665
622	1000+	-	329.77	1.19	665

Preparation: 5 µM 4-dye probe in Tris-HCl buffer (pH=8.0) (1 mL); Incubation: T=37 °C. All measurements were performed in three parallels. The corresponding average values and standard deviations are reported.

3. Biological assays

Trypsin cleavage

Figure S9: LC-MS analysis of the 4-dye probe cleaved by trypsin (5 ng/mL, 2 hours).

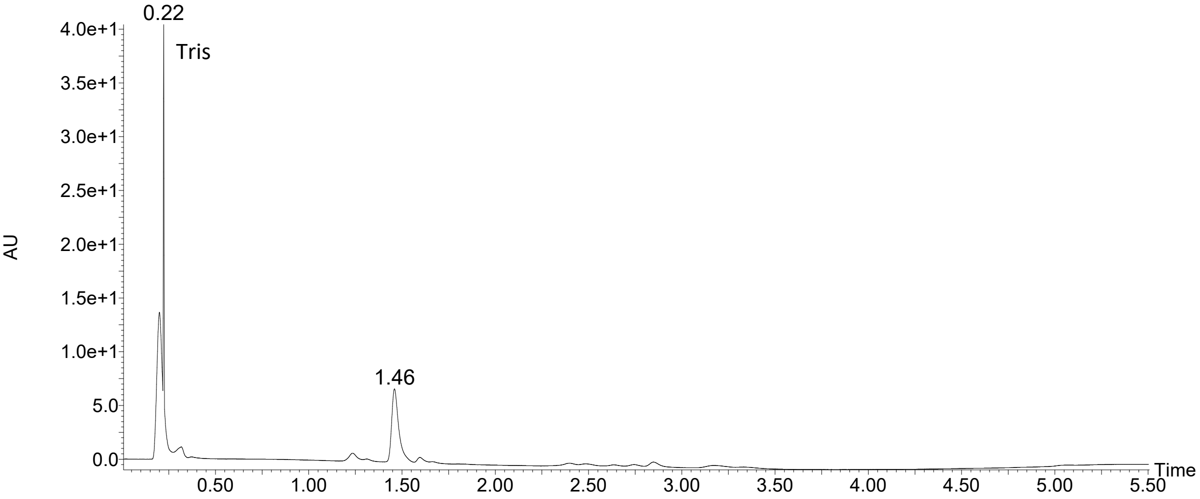

Fragment 1 (Rt = 1.46 min)

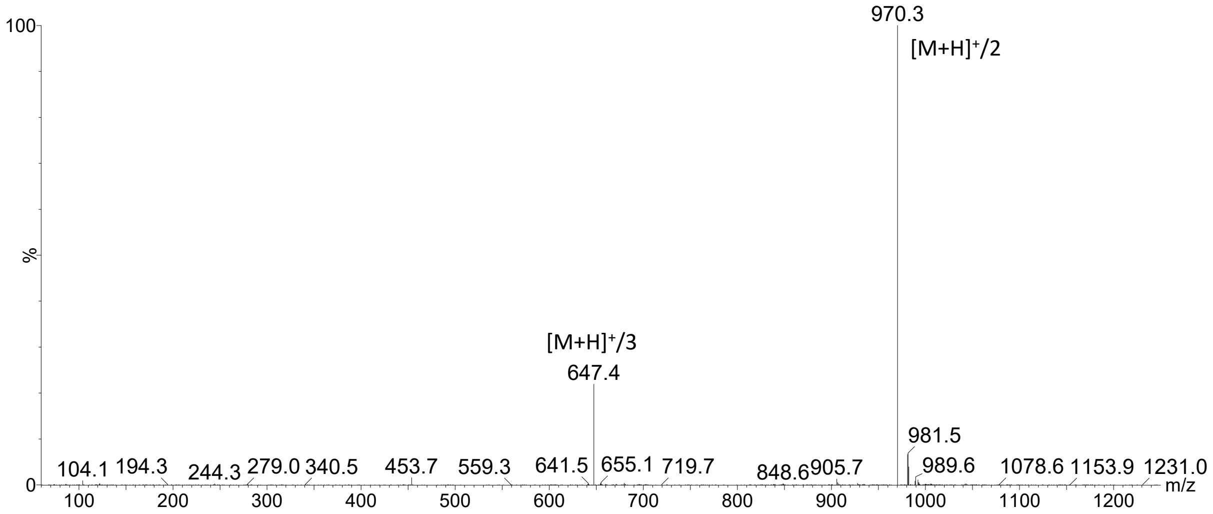

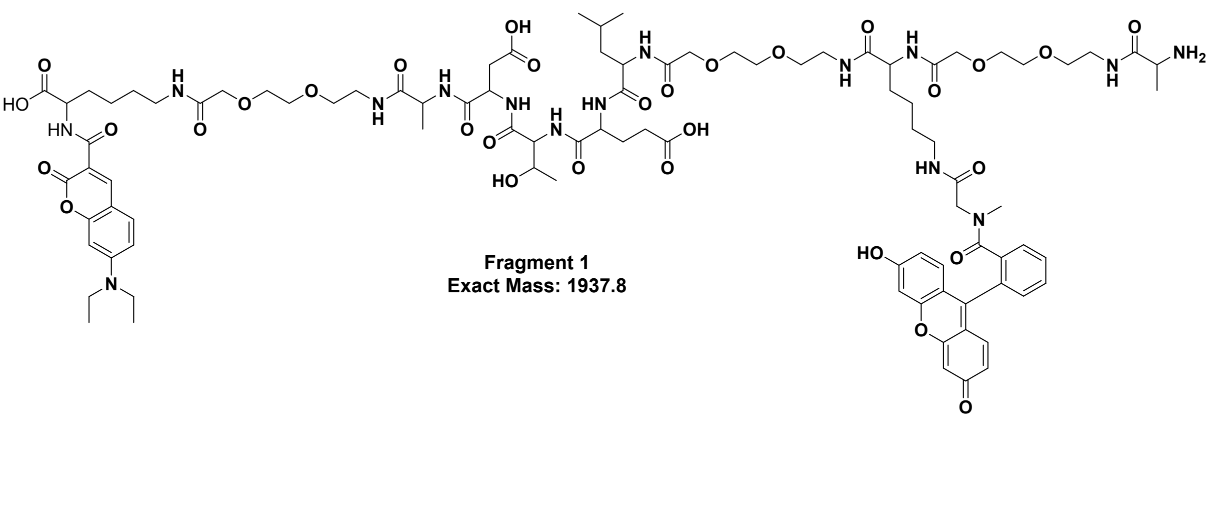

Chymotrypsin cleavage

Figure S10: LC-MS analysis of the 4-dye probe cleaved by chymotrypsin (2.5 µg/mL, 2 hours).

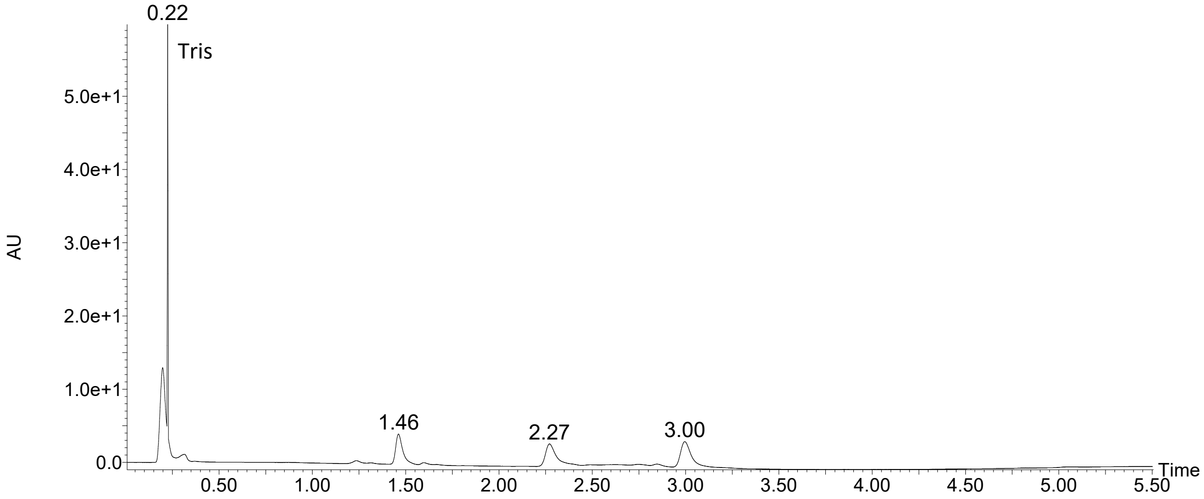

Fragment 1 (Rt = 1.46 min)

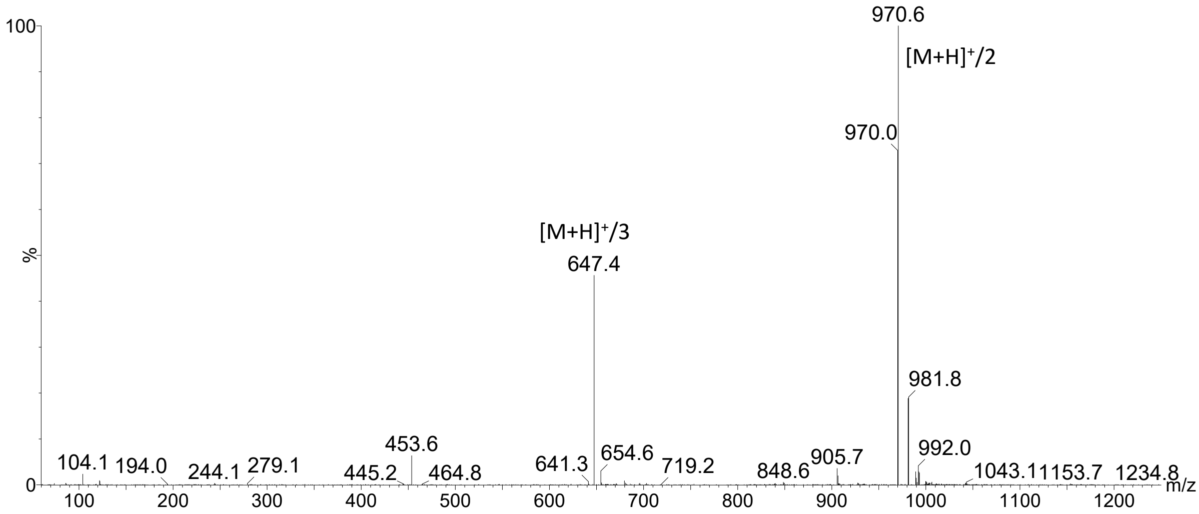

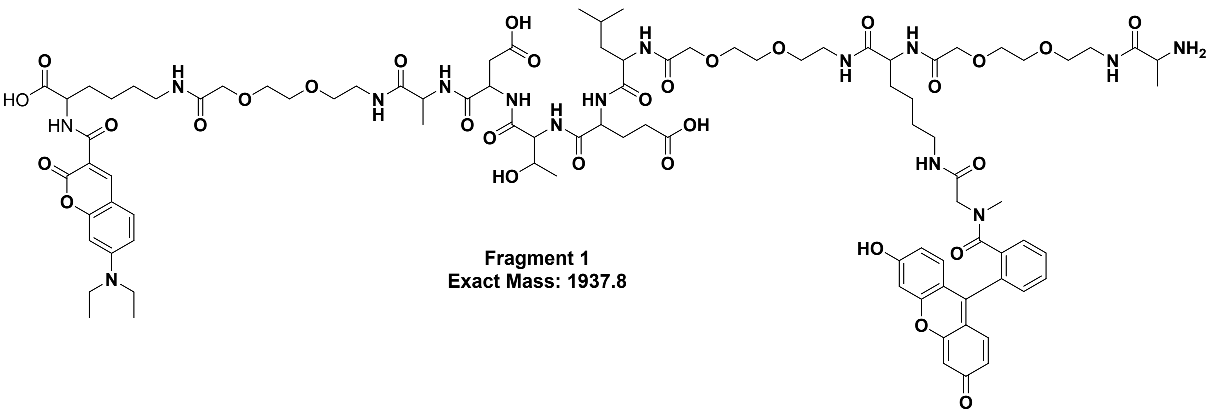

Fragment 2 (Rt = 2.27 min)

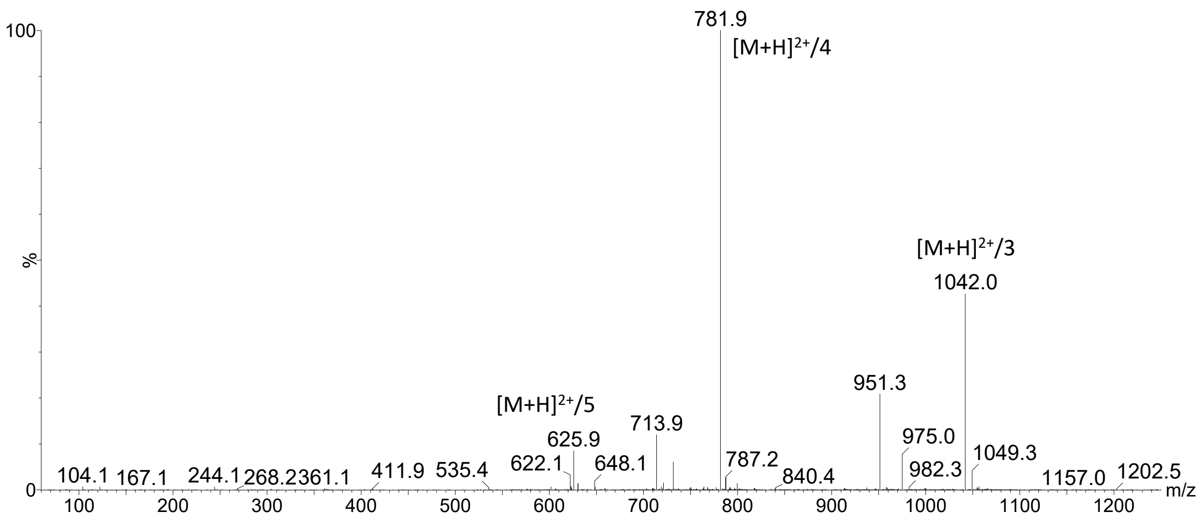

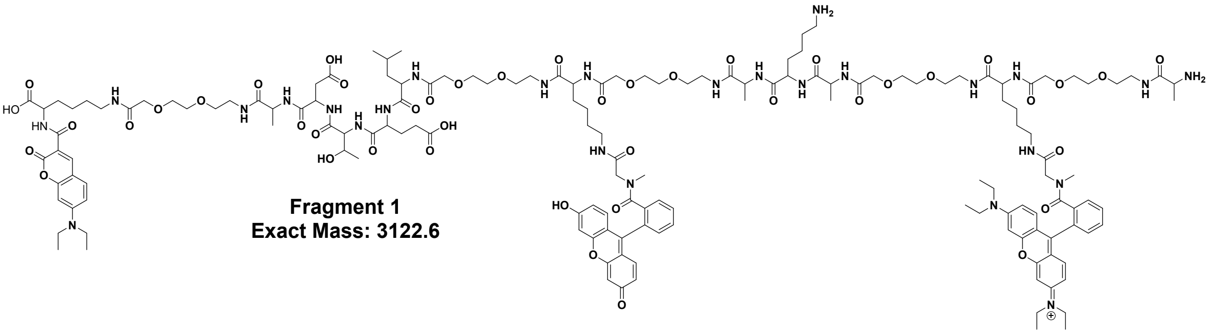

Fragment 1
Exact Mass: 3122.6

Fragment 3 (Rt = 3.00 min)

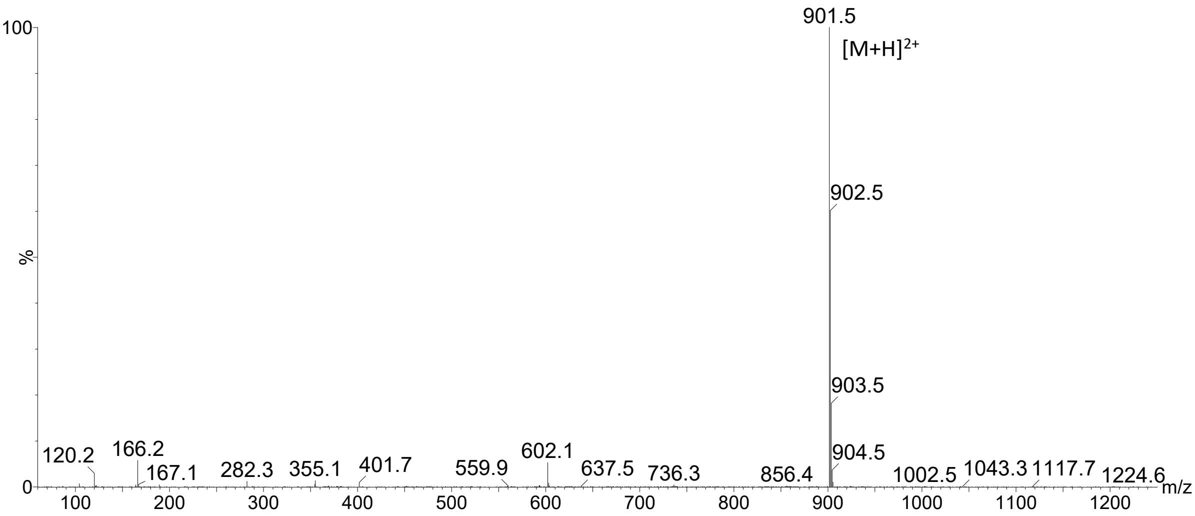

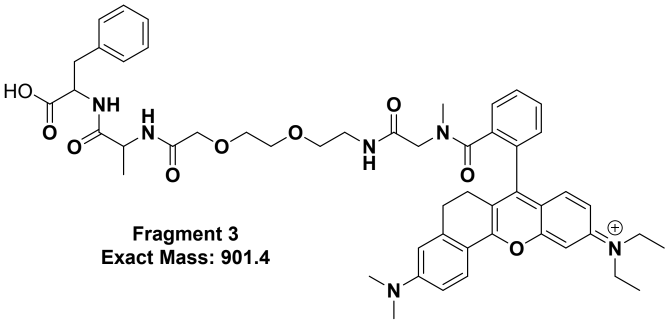

Fragment 3
Exact Mass: 901.4

Caspase-8 cleavage

Figure S11: LC-MS analysis of the 4-dye probe cleaved by caspase-8 (2 U/mL, 6 hours).

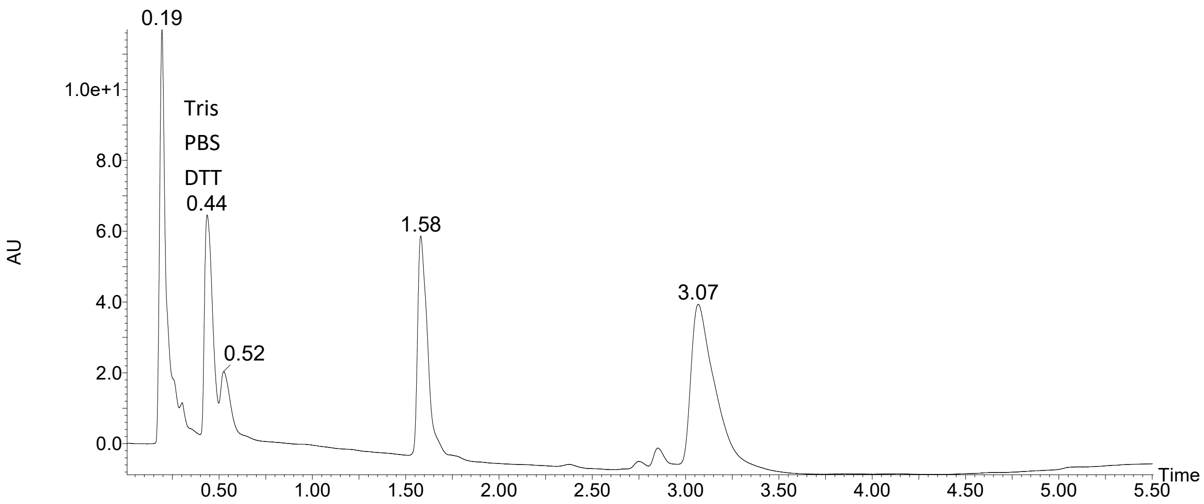

Fragment 1 (Rt = 1.58 min)

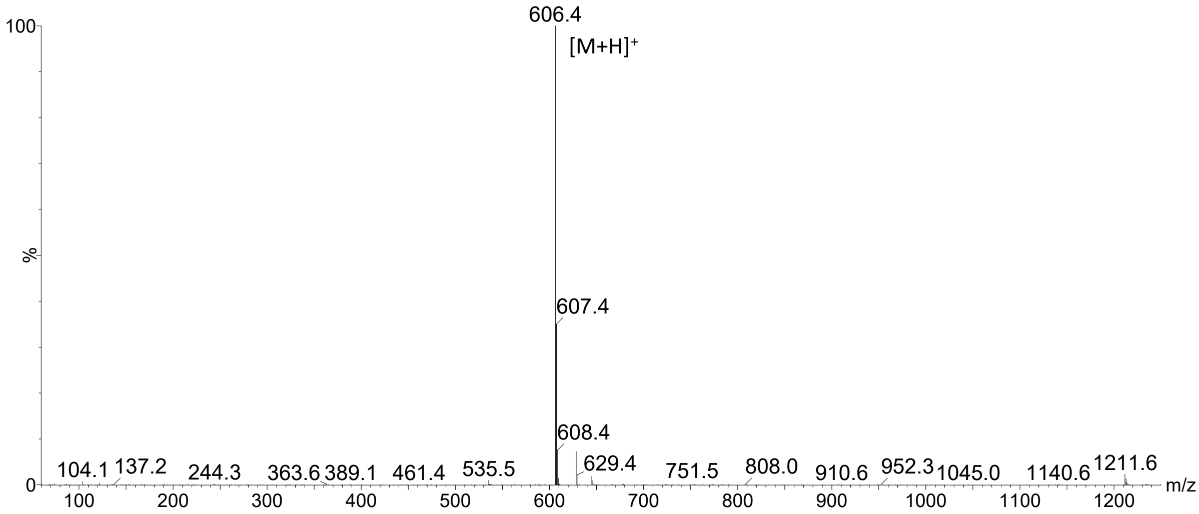

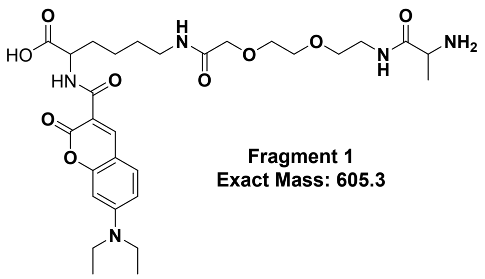

Fragment 1
Exact Mass: 605.3

Fragment 2 (Rt = 3.07 min)

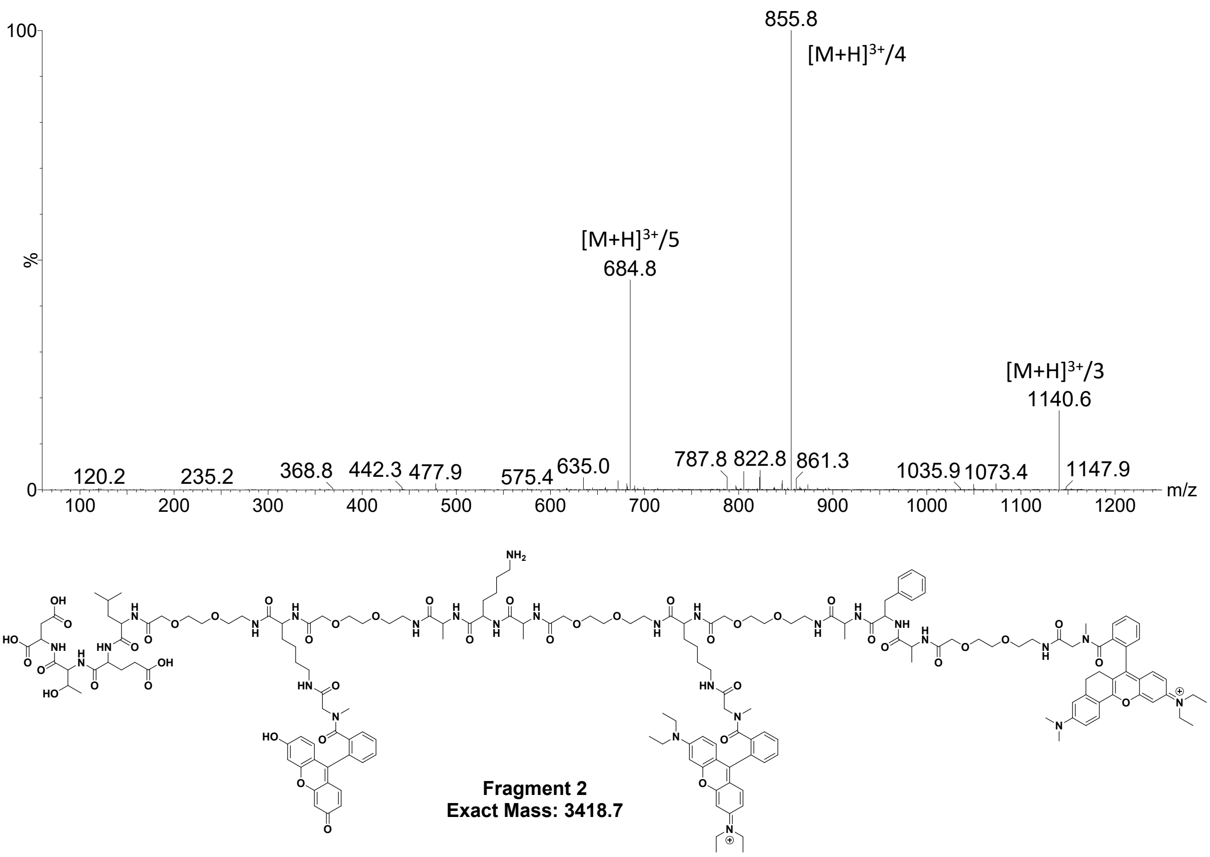

Figure S12: Characteristic cleavage patterns of the 4-dye probe in the presence of individual proteases.

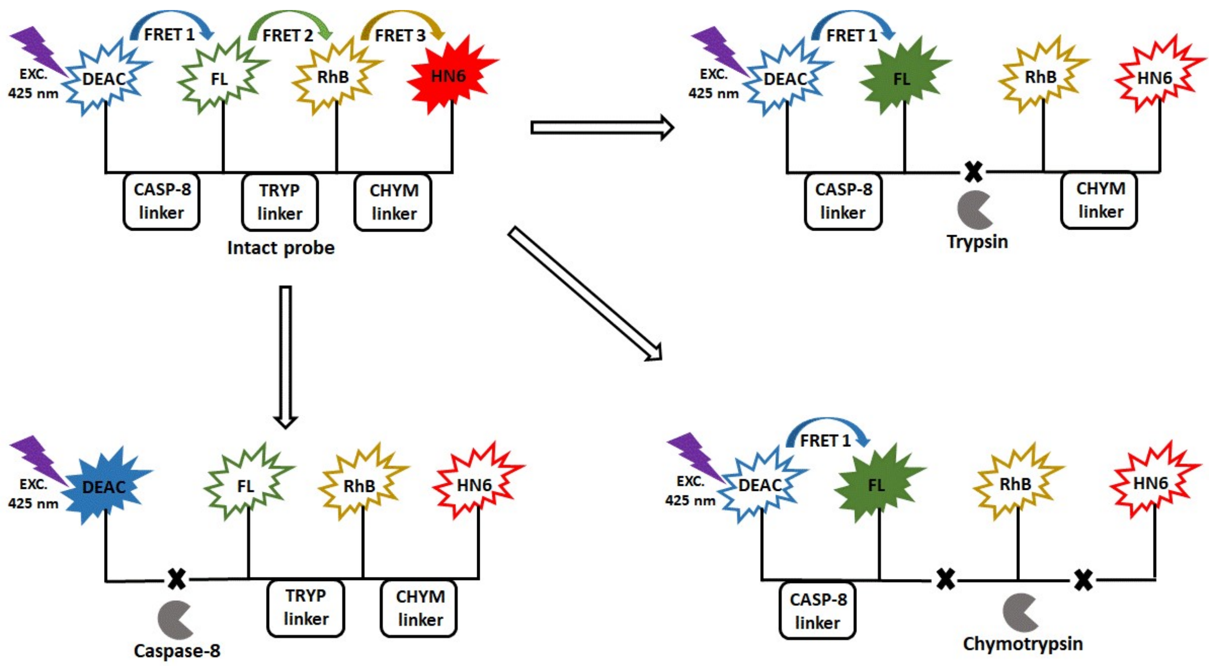

Table S5: Time-dependent fluorescence response of the 4-dye probe – blank sample.

Time [min]	DEAC Average	DEAC St. Dev.	FL Average	FL St. Dev.	RhB Average	RhB St. Dev.	HN6 Average	HN6 St. Dev.
0	20.32	0.47	14.65	0.46	10.76	0.35	181.64	2.56
2	20.61	0.49	14.78	0.37	10.63	0.18	176.17	1.27
5	20.69	0.65	14.94	0.31	10.83	0.26	176.01	0.71
8	20.75	0.46	15.08	0.41	10.75	0.18	175.65	0.46
12	20.75	0.32	15.03	0.30	10.81	0.17	175.94	0.97
16	20.94	0.36	15.19	0.31	11.00	0.26	176.52	0.31
20	20.95	0.22	15.33	0.28	11.09	0.15	176.65	1.22
25	21.09	0.40	15.28	0.15	11.02	0.17	175.90	1.41
30	21.27	0.43	15.54	0.20	11.10	0.20	176.32	0.74
35	21.10	0.49	15.63	0.27	11.32	0.23	176.60	0.83
40	21.05	0.51	15.97	0.23	11.39	0.31	176.34	1.01
45	21.36	0.58	15.80	0.40	11.54	0.17	176.84	0.85

Preparation: 5.3 µM 4-dye probe in Tris-HCl buffer (950 µL) (0 min), then addition of 0.9% saline (*w*/*V*) (30 µL) and 1 mM HCl (20 µL) (0–45 min); Incubation: T=37 °C; Excitation: λ=425 nm; Emissions: λ_{DEAC}=480 nm, λ_{FL}=522 nm, λ_{RhB}=595 nm, λ_{HN6}=665 nm; $Slit_{EXC}$/$Slit_{EMS}$=10/10 nm. All measurements were performed in three parallels. The corresponding average values and standard deviations are reported.

Table S6: Time-dependent fluorescence response of the 4-dye probe – detection of trypsin (c=0.5 ng/mL).

Time [min]	DEAC Average	DEAC St. Dev.	FL Average	FL St. Dev.	RhB Average	RhB St. Dev.	HN6 Average	HN6 St. Dev.
0	20.57	0.22	15.25	0.47	11.35	0.15	197.78	2.75
2	20.27	0.11	15.94	0.22	11.34	0.12	192.68	1.72
5	20.27	0.18	17.31	0.85	11.42	0.08	193.36	1.34
8	20.47	0.14	18.10	1.21	11.57	0.17	192.72	2.34
12	20.44	0.38	19.86	2.24	11.84	0.24	192.81	0.75
16	20.57	0.21	21.80	2.88	11.97	0.34	192.84	1.09
20	21.01	0.47	23.81	3.56	12.18	0.32	192.60	1.41
25	21.18	0.40	25.99	3.83	12.60	0.40	192.09	1.78
30	21.75	0.42	29.41	4.27	12.88	0.56	191.61	1.73
35	21.96	0.45	31.95	4.36	13.18	0.60	191.62	1.77
40	22.00	0.69	34.29	4.95	13.60	0.57	191.69	2.14
45	22.16	0.71	35.90	5.63	13.73	0.60	191.66	2.27

Preparation: 5.3 µM 4-dye probe in Tris-HCl buffer (950 µL) (0 min), then addition of 0.9% saline (*w*/*V*) (30 µL) and 1 mM HCl (20 µL) with trypsin (0.5 ng) (0–45 min); Incubation: T=37 °C; Excitation: λ=425 nm; Emissions: λ_{DEAC}=480 nm, λ_{FL}=522 nm, λ_{RhB}=595 nm, λ_{HN6}=665 nm; $Slit_{EXC}$/$Slit_{EMS}$=10/10 nm. All measurements were performed in three parallels. The corresponding average values and standard deviations are reported.

Table S7: Time-dependent fluorescence response of the 4-dye probe – detection of trypsin (c=1 ng/mL).

Time [min]	DEAC Average	DEAC St. Dev.	FL Average	FL St. Dev.	RhB Average	RhB St. Dev.	HN6 Average	HN6 St. Dev.
0	20.76	0.04	15.80	0.55	11.59	0.20	197.71	2.36
2	20.87	0.25	24.17	1.85	12.37	0.16	190.51	2.93
5	21.67	0.46	32.47	2.95	13.25	0.20	189.93	2.77
8	22.40	0.49	39.94	4.25	13.89	0.42	190.06	2.90
12	23.69	0.78	51.04	4.92	15.23	0.50	189.49	3.66
16	24.70	0.97	63.34	6.88	16.37	0.77	188.82	2.72
20	25.79	1.14	74.73	9.66	17.96	1.14	188.11	2.48
25	27.08	1.54	88.30	11.51	19.40	1.33	187.79	2.81
30	28.63	1.67	102.98	13.63	20.80	1.47	187.42	3.06
35	30.06	1.95	115.83	16.10	22.53	1.83	187.06	2.74
40	31.41	2.29	128.95	18.20	23.89	1.82	186.63	3.32
45	32.66	2.23	141.92	20.72	25.23	2.48	186.49	2.33

Preparation: 5.3 µM 4-dye probe in Tris-HCl buffer (950 µL) (0 min), then addition of 0.9% saline (*w*/*V*) (30 µL) and 1 mM HCl (20 µL) with trypsin (1 ng) (0–45 min); Incubation: T=37 °C; Excitation: λ=425 nm; Emissions: λ_{DEAC}=480 nm, λ_{FL}=522 nm, λ_{RhB}=595 nm, λ_{HN6}=665 nm; $Slit_{EXC}/Slit_{EMS}$=10/10 nm. All measurements were performed in three parallels. The corresponding average values and standard deviations are reported.

Table S8: Time-dependent fluorescence response of the 4-dye probe – detection of trypsin (c=5 ng/mL).

Time [min]	DEAC Average	DEAC St. Dev.	FL Average	FL St. Dev.	RhB Average	RhB St. Dev.	HN6 Average	HN6 St. Dev.
0	20.29	0.29	14.95	0.09	11.18	0.18	192.22	2.96
2	23.63	0.30	55.64	4.02	15.29	0.28	183.60	1.82
5	28.70	0.06	111.33	1.57	21.33	0.13	180.37	1.36
8	34.49	0.81	164.78	4.77	27.00	0.44	177.93	1.83
12	40.07	0.68	222.77	5.59	33.37	0.74	176.75	2.24
16	45.18	0.49	273.28	5.74	38.36	0.62	174.68	1.66
20	51.34	0.78	332.30	7.12	44.97	0.60	172.56	1.59
25	59.08	1.26	404.15	12.14	52.44	1.50	170.22	1.46
30	64.95	1.21	460.87	12.84	58.44	1.78	167.77	1.59
35	71.32	1.17	520.18	13.97	65.05	1.88	166.52	1.96
40	76.56	1.89	572.10	15.27	70.66	1.69	164.66	2.15
45	82.07	2.02	619.17	15.19	75.72	1.89	162.36	2.07

Preparation: 5.3 µM 4-dye probe in Tris-HCl buffer (950 µL) (0 min), then addition of 0.9% saline (*w*/*V*) (30 µL) and 1 mM HCl (20 µL) with trypsin (5 ng) (0–45 min); Incubation: T=37 °C; Excitation: λ=425 nm; Emissions: λ_{DEAC}=480 nm, λ_{FL}=522 nm, λ_{RhB}=595 nm, λ_{HN6}=665 nm; $Slit_{EXC}/Slit_{EMS}$=10/10 nm. All measurements were performed in three parallels. The corresponding average values and standard deviations are reported.

Table S9: Time-dependent fluorescence response of the 4-dye probe – detection of trypsin (c=10 ng/mL).

Time [min]	DEAC Average	DEAC St. Dev.	FL Average	FL St. Dev.	RhB Average	RhB St. Dev.	HN6 Average	HN6 St. Dev.
0	21.69	0.42	19.89	0.92	12.64	0.33	198.01	1.03
2	25.76	2.48	72.55	25.61	18.28	2.48	187.90	5.24
5	32.32	5.04	141.75	51.16	25.74	5.42	185.21	7.14
8	39.20	6.43	206.48	69.75	32.39	7.18	182.16	7.67
12	50.33	8.15	315.05	78.72	44.08	8.20	178.67	6.74
16	60.04	7.85	409.68	76.28	53.84	8.14	174.95	6.24
20	68.80	9.12	493.76	88.77	62.84	9.34	172.33	7.60
25	81.50	9.58	610.88	90.35	75.48	9.42	167.74	6.96
30	94.13	4.73	721.69	48.33	87.94	4.95	163.95	5.02
35	100.05	5.72	779.58	49.27	94.07	5.38	162.72	4.92
40	109.15	4.67	855.97	43.27	102.56	4.57	159.61	5.06
45	116.79	3.37	921.91	25.75	109.57	3.00	156.52	4.50

Preparation: 5.3 µM 4-dye probe in Tris-HCl buffer (950 µL) (0 min), then addition of 0.9% saline (*w*/*V*) (30 µL) and 1 mM HCl (20 µL) with trypsin (10 ng) (0–45 min); Incubation: T=37 °C; Excitation: λ=425 nm; Emissions: λ_{DEAC}=480 nm, λ_{FL}=522 nm, λ_{RhB}=595 nm, λ_{HN6}=665 nm; $Slit_{EXC}/Slit_{EMS}$=10/10 nm. All measurements were performed in three parallels. The corresponding average values and standard deviations are reported.

Table S10: Time-dependent fluorescence response of the 4-dye probe – detection of chymotrypsin (c=0.2 µg/mL).

Time [min]	DEAC Average	DEAC St. Dev.	FL Average	FL St. Dev.	RhB Average	RhB St. Dev.	HN6 Average	HN6 St. Dev.
0	20.59	0.33	15.81	0.48	11.50	0.13	196.97	2.16
2	20.39	0.44	16.90	0.39	12.44	0.10	193.70	1.20
5	20.46	0.27	18.80	0.48	13.69	0.29	195.66	1.57
8	20.97	0.33	20.87	0.58	14.96	0.14	197.00	1.86
12	21.45	0.35	24.20	0.94	17.10	0.25	200.13	1.86
16	21.86	0.34	26.88	0.86	19.01	0.34	202.51	1.47
20	22.18	0.18	29.62	0.63	20.42	0.04	205.75	2.26
25	22.88	0.23	33.03	0.81	22.80	0.32	208.57	2.13
30	23.37	0.33	37.08	0.96	25.28	0.23	211.94	2.13
35	24.02	0.33	40.96	0.95	27.34	0.48	215.55	2.08
40	24.71	0.36	44.10	0.70	29.01	0.52	219.03	2.22
45	25.02	0.27	47.26	0.36	31.21	0.32	221.28	1.90

Preparation: 5.3 µM 4-dye probe in Tris-HCl buffer (950 µL) (0 min), then addition of 0.9% saline (*w*/*V*) (30 µL) and 1 mM HCl (20 µL) with chymotrypsin (0.2 µg) (0–45 min); Incubation: T=37 °C; Excitation: λ=425 nm; Emissions: λ_{DEAC}=480 nm, λ_{FL}=522 nm, λ_{RhB}=595 nm, λ_{HN6}=665 nm; $Slit_{EXC}/Slit_{EMS}$=10/10 nm. All measurements were performed in three parallels. The corresponding average values and standard deviations are reported.

Table S11: Time-dependent fluorescence response of the 4-dye probe – detection of chymotrypsin (c=0.5 µg/mL).

Time [min]	DEAC Average	DEAC St. Dev.	FL Average	FL St. Dev.	RhB Average	RhB St. Dev.	HN6 Average	HN6 St. Dev.
0	20.42	0.15	16.36	0.47	11.67	0.33	191.07	2.28
2	20.33	0.31	19.90	0.86	14.32	0.66	191.68	1.17
5	21.30	0.40	24.98	2.02	18.06	1.44	195.74	2.01
8	21.96	0.35	30.01	2.77	21.18	1.89	201.06	2.94
12	22.81	0.37	36.31	2.70	25.19	2.02	207.63	2.37
16	24.01	0.30	42.84	1.88	29.16	1.43	213.78	1.17
20	25.24	0.20	50.86	1.23	33.97	0.97	219.63	1.58
25	26.96	0.47	62.21	3.00	40.32	1.44	229.22	1.52
30	28.05	0.39	68.70	1.11	44.16	0.24	235.66	1.10
35	29.23	0.42	75.68	1.28	47.87	1.33	241.55	1.32
40	30.45	0.10	83.21	1.83	52.45	1.13	248.20	0.91
45	31.99	0.49	91.59	0.82	56.58	0.47	254.47	1.77

Preparation: 5.3 µM 4-dye probe in Tris-HCl buffer (950 µL) (0 min), then addition of 0.9% saline (*w*/*V*) (30 µL) and 1 mM HCl (20 µL) with chymotrypsin (0.5 µg) (0–45 min); Incubation: T=37 °C; Excitation: λ=425 nm; Emissions: λ_{DEAC}=480 nm, λ_{FL}=522 nm, λ_{RhB}=595 nm, λ_{HN6}=665 nm; $Slit_{EXC}$/$Slit_{EMS}$=10/10 nm. All measurements were performed in three parallels. The corresponding average values and standard deviations are reported.

Table S12: Time-dependent fluorescence response of the 4-dye probe – detection of chymotrypsin (c=2.5 µg/mL).

Time [min]	DEAC Average	DEAC St. Dev.	FL Average	FL St. Dev.	RhB Average	RhB St. Dev.	HN6 Average	HN6 St. Dev.
0	20.00	0.25	15.00	0.46	11.11	0.27	188.50	1.53
2	22.87	0.54	35.38	0.73	25.56	0.51	209.12	1.19
5	26.95	0.61	61.83	3.00	42.20	1.48	232.15	2.25
8	30.75	0.55	85.77	0.96	55.22	0.46	253.12	2.18
12	34.79	0.50	114.37	1.98	69.31	0.61	272.17	1.04
16	38.27	0.32	138.00	0.29	79.79	0.20	289.31	2.46
20	42.25	0.13	165.23	2.11	91.31	1.19	303.75	2.10
25	46.88	0.16	199.86	2.23	103.41	1.07	321.57	1.69
30	51.37	0.56	231.66	1.94	114.20	0.60	335.35	0.68
35	54.99	0.39	259.15	1.13	121.96	0.44	346.15	0.90
40	58.91	0.38	286.81	1.37	129.38	0.65	355.28	0.85
45	62.62	0.59	314.24	0.89	135.91	0.57	363.24	0.55

Preparation: 5.3 µM 4-dye probe in Tris-HCl buffer (950 µL) (0 min), then addition of 0.9% saline (*w*/*V*) (30 µL) and 1 mM HCl (20 µL) with chymotrypsin (2.5 µg) (0–45 min); Incubation: T=37 °C; Excitation: λ=425 nm; Emissions: λ_{DEAC}=480 nm, λ_{FL}=522 nm, λ_{RhB}=595 nm, λ_{HN6}=665 nm; $Slit_{EXC}$/$Slit_{EMS}$=10/10 nm. All measurements were performed in three parallels. The corresponding average values and standard deviations are reported.

Table S13: Time-dependent fluorescence response of the 4-dye probe – detection of chymotrypsin (c=5.0 µg/mL).

Time [min]	DEAC Average	DEAC St. Dev.	FL Average	FL St. Dev.	RhB Average	RhB St. Dev.	HN6 Average	HN6 St. Dev.
0	19.97	0.19	15.16	0.32	11.12	0.18	191.20	0.63
2	24.80	0.12	48.12	1.16	33.49	1.08	224.33	1.63
5	31.17	0.93	91.13	4.27	57.16	2.48	257.58	4.48
8	36.47	1.68	126.99	11.52	73.05	5.28	282.02	9.12
12	44.24	1.98	185.45	13.47	96.33	4.67	314.02	5.72
16	52.17	1.04	242.57	12.77	114.18	4.08	337.88	4.10
20	59.64	0.55	298.06	3.70	129.31	1.54	357.36	2.32
25	67.42	1.06	361.30	8.13	143.85	0.70	374.64	2.17
30	73.48	1.07	404.65	8.59	152.79	2.83	383.94	1.62
35	78.88	1.63	447.71	11.80	160.01	2.49	391.16	1.82
40	84.68	0.54	494.37	5.29	167.92	2.03	398.78	2.05
45	88.85	0.68	532.79	6.60	174.01	1.14	404.13	0.91

Preparation: 5.3 µM 4-dye probe in Tris-HCl buffer (950 µL) (0 min), then addition of 0.9% saline (*w*/*V*) (30 µL) and 1 mM HCl (20 µL) with chymotrypsin (5.0 µg) (0–45 min); Incubation: T=37 °C; Excitation: λ=425 nm; Emissions: λ_{DEAC}=480 nm, λ_{FL}=522 nm, λ_{RhB}=595 nm, λ_{HN6}=665 nm; $Slit_{EXC}$/$Slit_{EMS}$=10/10 nm. All measurements were performed in three parallels. The corresponding average values and standard deviations are reported.

Table S14: Time-dependent fluorescence response of the 4-dye probe – detection of caspase-8 (c=2 U/mL).

Time [min]	DEAC Average	DEAC St. Dev.	FL Average	FL St. Dev.	RhB Average	RhB St. Dev.	HN6 Average	HN6 St. Dev.
0	20.57	0.42	16.70	0.10	14.58	0.49	190.39	1.03
2	21.60	0.51	16.87	0.20	15.06	0.08	184.60	0.87
5	22.82	0.70	17.39	0.32	15.33	0.21	184.10	1.33
8	24.49	0.76	18.25	0.20	15.65	0.19	182.36	1.50
12	27.10	1.40	19.20	0.67	16.18	0.25	182.28	1.20
16	30.15	1.94	20.29	0.63	16.67	0.19	181.56	1.63
20	33.42	2.57	21.17	0.81	16.91	0.24	180.95	1.25
25	36.27	2.98	22.27	0.86	17.37	0.17	181.78	2.02
30	39.26	3.65	23.35	1.24	17.84	0.08	180.27	1.80
35	41.90	3.77	24.16	1.00	18.06	0.13	180.05	1.91
40	44.56	3.75	25.17	1.16	18.49	0.03	179.74	2.60
45	47.04	4.33	26.00	1.24	18.55	0.23	180.21	1.79

Preparation: 5.3 µM 4-dye probe in Tris-HCl buffer (950 µL) with DTT (10 µmol) (0 min), then addition of 0.9% saline (*w*/*V*) (30 µL) with caspase-8 (2 U) and 1 mM HCl (20 µL) (0–45 min); Incubation: T=37 °C; Excitation: λ=425 nm; Emissions: λ_{DEAC}=480 nm, λ_{FL}=522 nm, λ_{RhB}=595 nm, λ_{HN6}=665 nm; $Slit_{EXC}$/$Slit_{EMS}$=10/10 nm. All measurements were performed in three parallels. The corresponding average values and standard deviations are reported.

Table S15: Time-dependent fluorescence response of the 4-dye probe – detection of caspase-8 (c=5 U/mL).

Time [min]	DEAC Average	DEAC St. Dev.	FL Average	FL St. Dev.	RhB Average	RhB St. Dev.	HN6 Average	HN6 St. Dev.
0	21.65	0.69	17.28	0.75	15.05	0.45	198.70	2.14
2	24.83	1.46	18.70	0.46	16.30	0.39	199.10	2.10
5	28.57	2.77	19.83	1.04	16.84	0.34	198.41	2.52
8	32.45	3.98	21.18	1.31	17.32	0.30	198.26	3.02
12	37.20	5.58	23.11	1.72	17.65	0.49	197.19	3.04
16	42.66	7.05	24.40	2.07	18.34	0.26	195.75	3.84
20	48.22	8.22	26.21	2.45	18.50	0.55	194.97	3.34
25	54.78	9.77	28.40	2.94	19.34	0.25	193.81	4.23
30	62.14	11.73	30.51	3.45	19.67	0.38	192.16	5.27
35	68.76	13.09	32.97	3.91	20.22	0.45	190.89	5.23
40	75.09	14.96	34.96	4.38	20.52	0.36	190.31	5.76
45	81.40	17.45	37.01	5.13	20.96	0.47	187.71	7.21

Preparation: 5.3 µM 4-dye probe in Tris-HCl buffer (950 µL) with DTT (10 µmol) (0 min), then addition of 0.9% saline (*w*/*V*) (30 µL) with caspase-8 (5 U) and 1 mM HCl (20 µL) (0–45 min); Incubation: T=37 °C; Excitation: λ=425 nm; Emissions: λ_{DEAC}=480 nm, λ_{FL}=522 nm, λ_{RhB}=595 nm, λ_{HN6}=665 nm; $Slit_{EXC}$/$Slit_{EMS}$=10/10 nm. All measurements were performed in three parallels. The corresponding average values and standard deviations are reported.

Table S16: Time-dependent fluorescence response of the 4-dye probe – detection of caspase-8 (c=10 U/mL).

Time [min]	DEAC Average	DEAC St. Dev.	FL Average	FL St. Dev.	RhB Average	RhB St. Dev.	HN6 Average	HN6 St. Dev.
0	21.42	0.55	17.63	0.48	16.19	1.15	200.51	5.00
2	30.10	0.44	20.39	0.32	16.96	0.97	195.07	5.15
5	40.61	1.24	23.69	0.83	17.62	1.14	192.02	4.68
8	51.38	2.11	26.95	0.95	18.13	1.11	189.46	4.18
12	64.38	3.21	31.06	1.21	18.70	1.00	185.51	3.83
16	77.46	4.34	35.01	1.58	19.35	1.06	182.77	3.37
20	91.38	6.14	38.80	2.52	20.06	0.87	179.62	2.96
25	104.81	8.13	43.17	3.08	20.71	0.96	176.13	1.16
30	118.87	10.74	47.26	3.47	21.20	0.96	173.35	0.79
35	129.60	11.34	50.43	3.96	21.86	1.07	170.92	0.19
40	139.80	13.57	53.59	4.38	22.38	0.84	168.35	1.09
45	149.69	14.25	56.78	4.37	22.64	0.85	165.48	1.00

Preparation: 5.3 µM 4-dye probe in Tris-HCl buffer (950 µL) with DTT (10 µmol) (0 min), then addition of 0.9% saline (*w*/*V*) (30 µL) with caspase-8 (10 U) and 1 mM HCl (20 µL) (0–45 min); Incubation: T=37 °C; Excitation: λ=425 nm; Emissions: λ_{DEAC}=480 nm, λ_{FL}=522 nm, λ_{RhB}=595 nm, λ_{HN6}=665 nm; $Slit_{EXC}$/$Slit_{EMS}$=10/10 nm. All measurements were performed in three parallels. The corresponding average values and standard deviations are reported.

Table S17: Time-dependent fluorescence response of the 4-dye probe – simultaneous screening of caspase-8 (c=2 U/mL) and trypsin (c=1 ng/mL) – addition of DTT after 20 min of the experiment.

Time [min]	DEAC Average	DEAC St. Dev.	FL Average	FL St. Dev.	RhB Average	RhB St. Dev.	HN6 Average	HN6 St. Dev.
0	20.71	0.70	16.17	0.42	11.79	0.15	194.18	3.10
2	26.03	0.69	25.20	1.66	12.47	0.15	188.96	1.47
5	35.57	1.64	38.03	3.75	13.85	0.37	185.67	1.29
8	44.42	1.77	49.95	6.94	15.24	0.81	181.68	2.43
12	58.97	3.52	64.84	9.59	16.73	1.17	178.30	2.00
16	71.45	3.64	78.06	12.03	18.25	1.42	175.21	1.89
20	84.91	3.16	91.19	14.69	19.63	1.60	171.71	2.77
25	99.40	2.01	93.47	12.90	20.69	1.45	162.76	2.80
30	110.03	3.52	95.31	13.16	21.85	1.42	160.58	2.68
35	119.11	5.53	96.15	12.37	22.47	1.42	157.66	3.98
40	128.06	5.08	98.16	11.91	23.00	1.38	156.04	3.38
45	137.54	4.19	99.72	11.67	23.79	1.55	153.69	3.52

Preparation: 5.3 µM 4-dye probe in Tris-HCl buffer (950 µL) (0 min), then addition of 0.9% saline (*w*/*V*) (30 µL) with caspase-8 (2U) and 1 mM HCl (20 µL) with trypsin (1 ng) (0–20 min), then addition of 200 mM DTT in Tris-HCl buffer (50 µL) (20–45 min); Incubation: T=37 °C; Excitation: λ=425 nm; Emissions: λ_{DEAC}=480 nm, λ_{FL}=522 nm, λ_{RhB}=595 nm, λ_{HN6}=665 nm; $Slit_{EXC}/Slit_{EMS}$=10/10 nm. All measurements were performed in three parallels. The corresponding average values and standard deviations are reported.

Table S18: Time-dependent fluorescence response of the 4-dye probe – screening of trypsin (c=1 ng/mL) – addition of DTT after 20 min of the experiment.

Time [min]	DEAC Average	DEAC St. Dev.	FL Average	FL St. Dev.	RhB Average	RhB St. Dev.	HN6 Average	HN6 St. Dev.
0	19.44	0.14	15.06	0.48	10.88	0.02	186.69	4.22
2	19.60	0.50	22.43	1.93	11.54	0.37	180.93	4.73
5	20.58	0.63	32.09	4.81	12.59	0.45	179.74	4.28
8	21.13	0.81	39.88	7.17	13.53	0.79	178.20	4.81
12	22.34	1.04	51.36	10.28	14.63	1.15	178.67	4.58
16	23.64	1.36	63.03	13.37	15.81	1.58	178.50	4.67
20	24.85	1.70	73.93	15.55	17.17	1.70	178.26	4.17
25	24.95	1.64	76.49	15.91	18.63	2.02	173.51	4.57
30	24.93	1.71	76.56	16.09	19.39	1.96	174.04	4.16
35	25.26	1.96	77.10	15.82	20.31	2.09	174.43	4.39
40	25.36	1.80	78.10	16.42	20.89	2.06	174.39	4.58
45	25.38	1.76	77.70	16.02	21.38	1.97	175.37	4.33

Preparation: 5.3 µM 4-dye probe in Tris-HCl buffer (950 µL) (0 min), then addition of 0.9% saline (*w*/*V*) (30 µL) and 1 mM HCl (20 µL) with trypsin (1 ng) (0–20 min), then addition of 200 mM DTT in Tris-HCl buffer (50 µL) (20–45 min); Incubation: T=37 °C; Excitation: λ=425 nm; Emissions: λ_{DEAC}=480 nm, λ_{FL}=522 nm, λ_{RhB}=595 nm, λ_{HN6}=665 nm; $Slit_{EXC}/Slit_{EMS}$=10/10 nm. All measurements were performed in three parallels. The corresponding average values and standard deviations are reported.

Table S19: Time-dependent fluorescence response of the 4-dye probe – simultaneous screening of caspase-8 (c=2 U/mL) and chymotrypsin (c=0.5 µg/mL) – addition of DTT after 20 min of the experiment.

Time [min]	DEAC Average	DEAC St. Dev.	FL Average	FL St. Dev.	RhB Average	RhB St. Dev.	HN6 Average	HN6 St. Dev.
0	20.28	0.25	15.22	0.26	11.23	0.15	198.18	4.66
2	21.50	0.43	19.55	0.47	13.98	0.19	193.83	1.75
5	23.59	0.36	25.14	0.34	17.41	0.38	196.61	1.92
8	26.10	0.46	30.55	0.52	20.68	0.15	200.71	1.00
12	29.40	0.93	37.91	0.84	25.06	0.37	205.93	1.85
16	33.65	1.43	45.50	0.80	29.28	0.38	210.91	1.43
20	37.60	1.86	51.71	0.77	33.03	0.31	216.68	0.97
25	39.83	1.74	55.24	0.97	36.97	0.40	214.26	1.05
30	42.69	1.19	56.06	0.57	39.70	0.41	217.24	1.45
35	45.63	2.10	56.86	0.97	41.68	0.56	217.34	1.62
40	48.47	2.10	57.80	0.91	42.56	0.38	218.61	1.68
45	50.65	2.85	59.06	1.27	43.16	0.53	218.67	1.64

Preparation: 5.3 µM 4-dye probe in Tris-HCl buffer (950 µL) (0 min), then addition of 0.9% saline (*w*/*V*) (30 µL) with caspase-8 (2U) and 1 mM HCl (20 µL) with chymotrypsin (0.5 µg) (0–20 min), then addition of 200 mM DTT in Tris-HCl buffer (50 µL) (20–45 min); Incubation: T=37 °C; Excitation: λ=425 nm; Emissions: λ_{DEAC}=480 nm, λ_{FL}=522 nm, λ_{RhB}=595 nm, λ_{HN6}=665 nm; $Slit_{EXC}/Slit_{EMS}$=10/10 nm. All measurements were performed in three parallels. The corresponding average values and standard deviations are reported.

Table S20: Time-dependent fluorescence response of the 4-dye probe – screening of chymotrypsin (c=0.5 µg/mL) – addition of DTT after 20 min of the experiment.

Time [min]	DEAC Average	DEAC St. Dev.	FL Average	FL St. Dev.	RhB Average	RhB St. Dev.	HN6 Average	HN6 St. Dev.
0	19.45	0.33	16.20	1.87	10.80	0.32	182.41	1.13
2	19.84	0.35	20.69	1.85	13.72	0.33	182.83	1.50
5	20.83	0.30	25.54	2.15	17.01	0.36	187.46	1.31
8	21.65	0.42	30.78	2.26	20.15	0.34	192.13	1.72
12	22.40	0.52	37.13	2.40	24.00	0.43	197.54	1.93
16	23.98	0.32	44.32	2.87	27.95	0.71	203.16	1.51
20	24.72	0.41	50.67	2.99	31.95	0.81	208.60	1.94
25	25.19	0.43	52.93	2.45	35.85	0.78	208.15	2.40
30	25.54	0.55	53.87	2.53	37.77	0.76	209.56	2.38
35	25.59	0.31	54.48	2.91	39.53	0.67	211.45	3.69
40	26.10	0.35	55.21	2.78	40.75	0.87	213.39	2.83
45	26.44	0.48	55.32	2.98	41.48	1.34	214.60	3.31

Preparation: 5.3 µM 4-dye probe in Tris-HCl buffer (950 µL) (0 min), then addition of 0.9% saline (*w*/*V*) (30 µL) and 1 mM HCl (20 µL) with chymotrypsin (0.5 µg) (0–20 min), then addition of 200 mM DTT in Tris-HCl buffer (50 µL) (20–45 min); Incubation: T=37 °C; Excitation: λ=425 nm; Emissions: λ_{DEAC}=480 nm, λ_{FL}=522 nm, λ_{RhB}=595 nm, λ_{HN6}=665 nm; $Slit_{EXC}/Slit_{EMS}$=10/10 nm. All measurements were performed in three parallels. The corresponding average values and standard deviations are reported.

Table S21: Time-dependent fluorescence response of the 4-dye probe – simultaneous screening of trypsin (c=1 ng/mL) and chymotrypsin (c=0.5 µg/mL) – addition of DTT after 20 min of the experiment.

Time [min]	DEAC Average	DEAC St. Dev.	FL Average	FL St. Dev.	RhB Average	RhB St. Dev.	HN6 Average	HN6 St. Dev.
0	20.29	0.62	15.56	0.15	11.28	0.22	189.25	4.23
2	20.98	0.44	25.65	2.01	14.54	0.56	188.33	3.66
5	22.75	0.97	40.29	4.87	18.90	1.38	192.37	2.79
8	24.18	1.02	52.55	7.98	22.98	1.50	196.19	2.33
12	26.73	0.87	70.48	10.84	29.19	0.85	203.39	3.33
16	28.77	1.24	87.14	13.12	34.62	0.74	210.46	5.64
20	30.81	1.32	104.67	15.24	39.77	1.15	215.78	3.69
25	31.43	1.56	108.16	16.71	43.15	1.92	215.00	3.34
30	31.96	1.67	109.33	16.34	46.10	1.86	218.12	4.71
35	31.93	1.79	109.76	16.03	47.65	1.88	219.42	3.98
40	32.21	1.78	110.38	16.11	48.55	1.87	220.53	4.10
45	32.49	1.48	110.92	15.98	49.89	1.79	222.65	4.01

Preparation: 5.3 µM 4-dye probe in Tris-HCl buffer (950 µL) (0 min), then addition of 0.9% saline (*w*/*V*) (30 µL) and 1 mM HCl (20 µL) with trypsin (1 ng) and chymotrypsin (c=0.5 µg) (0–20 min), then addition of 200 mM DTT in Tris-HCl buffer (50 µL) (20–45 min); Incubation: T=37 °C; Excitation: λ=425 nm; Emissions: λ_{DEAC}=480 nm, λ_{FL}=522 nm, λ_{RhB}=595 nm, λ_{HN6}=665 nm; $Slit_{EXC}/Slit_{EMS}$=10/10 nm. All measurements were performed in three parallels. The corresponding average values and standard deviations are reported.

Table S22: Time-dependent fluorescence response of the 4-dye probe – simultaneous screening of trypsin (c=1 ng/mL) and chymotrypsin (c=5.0 µg/mL) – addition of DTT after 20 min of the experiment.

Time [min]	DEAC Average	DEAC St. Dev.	FL Average	FL St. Dev.	RhB Average	RhB St. Dev.	HN6 Average	HN6 St. Dev.
0	20.17	0.50	15.35	0.52	11.08	0.28	189.39	2.07
2	26.36	0.79	58.38	1.29	38.36	0.56	229.88	2.89
5	33.72	1.04	110.86	2.89	63.72	1.10	265.87	2.91
8	40.58	1.08	159.30	5.08	84.13	1.18	292.62	4.69
12	48.44	0.91	219.17	4.69	104.39	1.06	319.07	3.88
16	55.52	0.72	274.44	3.52	120.50	0.78	340.11	4.49
20	62.10	0.75	327.30	9.04	133.43	1.56	354.25	3.90
25	63.82	0.98	333.93	9.21	138.60	0.85	351.10	3.77
30	64.39	1.53	335.69	8.72	141.84	1.76	357.67	5.34
35	65.32	1.32	337.51	9.16	144.44	1.39	359.05	4.64
40	65.23	1.42	337.86	8.89	145.66	0.62	361.54	4.44
45	66.15	1.16	338.09	9.45	147.00	1.16	361.47	4.54

Preparation: 5.3 µM 4-dye probe in Tris-HCl buffer (950 µL) (0 min), then addition of 0.9% saline (*w*/*V*) (30 µL) and 1 mM HCl (20 µL) with trypsin (1 ng) and chymotrypsin (c=5.0 µg) (0–20 min), then addition of 200 mM DTT in Tris-HCl buffer (50 µL) (20–45 min); Incubation: T=37 °C; Excitation: λ=425 nm; Emissions: λ_{DEAC}=480 nm, λ_{FL}=522 nm, λ_{RhB}=595 nm, λ_{HN6}=665 nm; $Slit_{EXC}/Slit_{EMS}$=10/10 nm. All measurements were performed in three parallels. The corresponding average values and standard deviations are reported.

Table S23: Time-dependent fluorescence response of the 4-dye probe – simultaneous screening of trypsin (c=10 ng/mL) and chymotrypsin (c=0.5 µg/mL) – addition of DTT after 20 min of the experiment.

Time [min]	DEAC Average	DEAC St. Dev.	FL Average	FL St. Dev.	RhB Average	RhB St. Dev.	HN6 Average	HN6 St. Dev.
0	20.05	0.38	15.75	0.77	11.19	0.34	191.89	5.12
2	28.81	0.41	110.43	1.12	23.52	0.11	185.08	4.35
5	40.51	0.38	224.26	3.83	38.37	0.47	186.70	4.95
8	51.08	0.28	327.49	5.18	52.19	0.77	188.47	5.02
12	64.05	0.53	447.75	8.38	67.80	1.09	190.83	4.63
16	75.18	1.19	555.14	10.40	82.13	1.30	191.95	3.92
20	85.67	0.77	644.21	14.35	94.22	1.58	195.00	4.95
25	86.88	1.24	652.02	16.82	98.15	2.18	193.13	4.76
30	86.78	1.03	653.70	16.26	100.12	1.74	195.45	5.03
35	86.71	1.51	653.36	16.91	101.81	1.98	198.24	4.96
40	87.55	1.12	654.70	17.15	102.76	2.42	199.06	4.96
45	87.39	1.30	654.15	16.34	103.73	2.04	200.34	5.49

Preparation: 5.3 µM 4-dye probe in Tris-HCl buffer (950 µL) (0 min), then addition of 0.9% saline (*w*/*V*) (30 µL) and 1 mM HCl (20 µL) with trypsin (10 ng) and chymotrypsin (c=0.5 µg) (0–20 min), then addition of 200 mM DTT in Tris-HCl buffer (50 µL) (20–45 min); Incubation: T=37 °C; Excitation: λ=425 nm; Emissions: λ_{DEAC}=480 nm, λ_{FL}=522 nm, λ_{RhB}=595 nm, λ_{HN6}=665 nm; $Slit_{EXC}/Slit_{EMS}$=10/10 nm. All measurements were performed in three parallels. The corresponding average values and standard deviations are reported.

Table S24: Ratios of fluorescence intensities of the 4-dye probe at times 45 min and 0 min.

Type of protease	Conc. [/mL]	DEAC (I_{45}/I_0)	DEAC St. Dev.	FL (I_{45}/I_0)	FL St. Dev.	DEAC(I_{45}/I_0) /FL(I_{45}/I_0)	DEAC/FL St. Dev.	HN6 (I_{45}/I_0)	HN6 St. Dev.
/	0	1.05	0.04	1.08	0.04	0.97	0.05	0.97	0.01
Tryp	0.5 ng	1.08	0.04	2.35	0.38	0.46	0.07	0.97	0.02
Tryp	1 ng	1.57	0.11	8.98	1.35	0.18	0.03	0.94	0.02
Tryp	5 ng	4.04	0.12	41.42	1.05	0.10	0.00	0.84	0.02
Tryp	10 ng	5.38	0.19	46.35	2.50	0.12	0.01	0.79	0.02
Chym	0.2 µg	1.22	0.02	2.99	0.09	0.41	0.01	1.12	0.02
Chym	0.5 µg	1.57	0.03	5.60	0.17	0.28	0.01	1.33	0.02
Chym	2.5 µg	3.13	0.05	20.95	0.65	0.15	0.01	1.93	0.02
Chym	5 µg	4.45	0.05	35.14	0.86	0.13	0.00	2.11	0.01
Casp-8	2 U	2.29	0.22	1.56	0.07	1.47	0.16	0.95	0.01
Casp-8	5 U	3.76	0.81	2.14	0.31	1.76	0.46	0.94	0.04
Casp-8	10 U	6.99	0.69	3.22	0.26	2.17	0.28	0.83	0.02

I_{45}/I_0 – ratio of fluorescence intensities of different fluorophores at times 45 min and 0 min;
St. Dev. – standard deviation of two data sets calculated by the formula $\Delta z=z*[(\Delta x/x)^2+(\Delta y/y)^2]^{1/2}$, where x, y, and z represent appropriate average values of individual data sets, while Δx, Δy, and Δz denote corresponding standard deviations.

Table S25: General criteria for individual proteases recognition after 45 min of the experiment.

Protease	X = DEAC(I_{45}/I_0)/FL(I_{45}/I_0)	Y = HN6(I_{45}/I_0)
/	0.75 < X < 1.25	Y < 1.05
Trypsin	X < 0.75	Y < 1.05
Chymotrypsin	X < 0.75	Y > 1.05
Caspase-8	X > 1.25	Y < 1.05

I_{45}/I_0 – ratio of fluorescence intensities of different fluorophores at times 45 min and 0 min.

Table S26: Ratios of fluorescence intensities of the 4-dye probe at times 16 min and 0 min.

Type of protease	Conc. [/mL]	DEAC (I_{16}/I_0)	DEAC St. Dev.	FL (I_{16}/I_0)	FL St. Dev.	DEAC(I_{16}/I_0) /FL(I_{16}/I_0)	DEAC/FL St. Dev.	HN6 (I_{16}/I_0)	HN6 St. Dev.
/	0	1.03	0.03	1.04	0.04	0.99	0.05	0.97	0.01
Tryp	0.5 ng	1.00	0.01	1.43	0.19	0.70	0.10	0.98	0.01
Tryp	1 ng	1.19	0.05	4.01	0.46	0.30	0.04	0.96	0.02
Tryp	5 ng	2.23	0.04	18.28	0.40	0.12	0.00	0.91	0.02
Tryp	10 ng	2.77	0.37	20.60	3.95	0.13	0.03	0.88	0.03
Chym	0.2 µg	1.06	0.02	1.70	0.07	0.62	0.03	1.03	0.01
Chym	0.5 µg	1.18	0.02	2.62	0.14	0.45	0.02	1.12	0.01
Chym	2.5 µg	1.91	0.03	9.20	0.28	0.21	0.01	1.53	0.02
Chym	5 µg	2.61	0.06	16.00	0.91	0.16	0.01	1.77	0.02
Casp-8	2 U	1.47	0.10	1.21	0.04	1.21	0.09	0.95	0.01
Casp-8	5 U	1.97	0.33	1.41	0.13	1.40	0.27	0.99	0.02
Casp-8	10 U	3.62	0.22	1.99	0.10	1.82	0.15	0.91	0.03

I_{16}/I_0 – ratio of fluorescence intensities of different fluorophores at times 16 min and 0 min;
St. Dev. – standard deviation of two data sets calculated by the formula $\Delta z = z*[(\Delta x/x)^2+(\Delta y/y)^2]^{1/2}$, where x, y, and z represent appropriate average values of individual data sets, while Δx, Δy, and Δz denote corresponding standard deviations.

Table S27: General criteria for individual proteases recognition after 16 min of the experiment.

Protease	X = DEAC(I_{16}/I_0)/FL(I_{16}/I_0)	Y = HN6(I_{16}/I_0)
/	0.90 < X < 1.10	Y < 1.00
Trypsin	X < 0.90	Y < 1.00
Chymotrypsin	X < 0.70	Y > 1.00
Caspase-8	X > 1.10	Y < 1.05

I_{16}/I_0 – ratio of fluorescence intensities of different fluorophores at times 16 min and 0 min.
